# Ethnic Disparities in COVID-19 Vaccine Mistrust and Receipt in British Columbia, Canada: Population Survey

**DOI:** 10.2196/48466

**Published:** 2024-02-16

**Authors:** Bushra Mahmood, Prince Adu, Geoffrey McKee, Aamir Bharmal, James Wilton, Naveed Zafar Janjua

**Affiliations:** 1 Division of Endocrinology University of British Columbia Vancouver, BC Canada; 2 British Columbia Center for Disease Control Vancouver, BC Canada; 3 Department of Social Medicine Heritage College of Osteopathic Medicine Ohio University Dublin, OH United States; 4 School of Population and Public Health University of British Columbia Vancouver, BC Canada; 5 Centre for Health Evaluation and Outcome Sciences St Paul’s Hospital Vancouver, BC Canada

**Keywords:** COVID-19, vaccine hesitancy, mistrust, trust, ethnic minorities, South Asian, vaccine, vaccination, hesitancy, ethnic, ethnicity, minority, cultural, racial, minorities, SARS-CoV-2, coronavirus, Asia, Asian

## Abstract

**Background:**

Racialized populations in the United States, Canada, and the United Kingdom have been disproportionately affected by COVID-19. Higher vaccine hesitancy has been reported among racial and ethnic minorities in some of these countries. In the United Kingdom, for example, higher vaccine hesitancy has been observed among the South Asian population and Black compared with the White population, and this has been attributed to lack of trust in government due to historical and ongoing racism and discrimination.

**Objective:**

This study aimed to assess vaccine receipt by ethnicity and its relationship with mistrust among ethnic groups in British Columbia (BC), Canada.

**Methods:**

We included adults ≥18 years of age who participated in the BC COVID-19 Population Mixing Patterns Survey (BC-Mix) from March 8, 2021, to August 8, 2022. The survey included questions about vaccine receipt and beliefs based on a behavioral framework. Multivariable logistic regression was used to assess the association between mistrust in vaccines and vaccine receipt among ethnic groups.

**Results:**

The analysis included 25,640 adults. Overall, 76.7% (22,010/28,696) of respondents reported having received at least 1 dose of COVID-19 vaccines (Chinese=86.1%, South Asian=79.6%, White=75.5%, and other ethnicity=73.2%). Overall, 13.7% (3513/25,640) of respondents reported mistrust of COVID-19 vaccines (Chinese=7.1%, South Asian=8.2%, White=15.4%, and other ethnicity=15.2%). In the multivariable model (adjusting for age, sex, ethnicity, educational attainment, and household size), mistrust was associated with a 93% reduced odds of vaccine receipt (adjusted odds ratio 0.07, 95% CI 0.06-0.08). In the models stratified by ethnicity, mistrust was associated with 81%, 92%, 94%, and 95% reduced odds of vaccine receipt among South Asian, Chinese, White, and other ethnicities, respectively. Indecision, whether to trust the vaccine or not, was significantly associated with a 70% and 78% reduced odds of vaccine receipt among those who identified as White and of other ethnic groups, respectively.

**Conclusions:**

Vaccine receipt among those who identified as South Asian and Chinese in BC was higher than that among the White population. Vaccine mistrust was associated with a lower odds of vaccine receipt in all ethnicities, but it had a lower effect on vaccine receipt among the South Asian and Chinese populations. Future research needs to focus on sources of mistrust to better understand its potential influence on vaccine receipt among visible minorities in Canada.

## Introduction

Vaccines are a successful, evidence-based measure against many infectious diseases and a key response to contain the spread of SARS-CoV-2 and prevent severe outcomes such as hospitalizations and death. Soon after the approval of COVID-19 vaccines by Health Canada, nationwide vaccination began on December 14, 2020. As of March 2023, approximately 87.1% of the Canadian population (aged 5 years and older) had received at least 1 dose of COVID-19 vaccines, and 84.5% had completed the 2-dose primary series of vaccination [1]. Although these numbers are impressive, vaccine receipt in Canada has varied by subgroups with some expressing more resistance or hesitancy [2]. Vaccine hesitancy, characterized as a “delay in acceptance or refusal of vaccines despite availability of vaccination services” [3], is a major barrier to prevent severe outcomes for SARS-CoV-2 infection.

The analysis of COVID-19 cases and mortality rates shows disproportionate infections and deaths across various racial and ethnic populations in many countries, including the United Kingdom, the United States, and Canada [4-6]. In the United Kingdom, despite an attenuation of the elevated risk of COVID-19 mortality after adjusting for sociodemographic characteristics and health status, risk remained substantially higher among South Asian people from Bangladeshi and Pakistani background in both the first and the second waves of the pandemic [5]. Similarly, in the United States, the African American or Black and Hispanic populations experienced disproportionately higher rates of SARS-CoV-2 infection and COVID-19–related mortality [4]. In Canada, the South Asian population, one of the fastest growing ethnic minorities [7], has been disproportionately impacted by the pandemic with an increased risk of both infection and mortality from COVID-19 [6]. Age-standardized mortality rates among the South Asian population (31 deaths per 100,000 population) were second to Black (49 deaths per 100,000 population), followed by Chinese (22 deaths per 100,000 population) [6]. Studies from the United Kingdom have also identified the South Asian population as one of the most affected groups with a 10%-50% higher risk of death related to COVID-19 compared with the White British population [8]. However, despite this elevated risk, studies have reported significantly low levels of vaccine acceptance among this group in the United Kingdom [9,10]. Gauging current levels of vaccine acceptance across various population groups (specifically those at higher risk of infection and severe outcomes) and identifying modifiable factors to increase vaccine receipt are important considerations to control COVID-19 [11] and reduce mortality and morbidity. Numerous studies have evaluated COVID-19 vaccine hesitancy among the general population. These studies have attributed various factors to low acceptance of vaccine including concerns around safety and effectiveness of the vaccine [12], potential side effects, and short duration of immunity [13]. Other factors include a low socioeconomic status, identifying as a member of a marginalized population (such as Black, Hispanic, Indigenous, or other person of color), and dissatisfaction or mistrust of the system based on past experiences of discrimination and systematic racism [14,15].

The rapid development of COVID-19 vaccines has been possible due to substantial public funding and unprecedented levels of scientific collaboration and innovation to address global public needs. However, building trust in the vaccine to encourage its receipt has been an uphill task for many governments and public health agencies [16]. Trust is defined as one’s belief that another person or institution will act in accordance with one’s expectations of positive behavior by others [17], and institutional trust is recognized as a key measure of government performance [18]. Some early studies on vaccine hesitancy implicated a general mistrust in the benefits and safety of vaccines and concerns about their unforeseen effect [19,20]. Medical mistrust is a key factor that underlies general health inequities [21] and pertains to both the absence of trust and a sense that someone or something is acting against one’s best interests; this can include health care providers, systems, and government [22,23]. A study investigating the role of medical mistrust in receipt of vaccines among ethnically diverse communities in the United Kingdom found medical mistrust to be associated with COVID-19 vaccine hesitancy and lower intention to vaccinate. Suspicion related to the benefits and safety of vaccines and concerns about their unforeseen effect were the main contributor to this relationship [21].

In this study, we investigated COVID-19 vaccine receipt and its relationship with mistrust in vaccines by ethnicity in British Columbia (BC), Canada.

## Methods

### Design

We used data from the BC COVID-19 Population Mixing Patterns Survey (BC-Mix), a repeated web-based survey, designed and launched to assess population mixing patterns in BC during the COVID-19 pandemic [24].

To capture participants from a broad demographic range, the survey invitation was disseminated through Instagram, Facebook, YouTube, WhatsApp, Twitter, and Google search engine results pages. The Google Ads Audience manager and Facebook Ads manager allow for paid advertisements to be targeted at specific audiences. We used these tools to target the survey advertisement campaigns to only residents of BC who are 18 years and older. To help capture underrepresented groups, we promoted the survey to various ethnic populations. For instance, a South Asian community organization promoted the survey on their social media pages and also sent the survey to individuals on their mailing address. Although the survey was in English, it was also promoted in different languages (specifically, Korean and Farsi) to members of minority community groups in BC on their social media pages. Flyers were also distributed at grocery stores and restaurants particularly including those frequented by minority groups.

As of August 30, 2022, over 94,000 participants had participated in the survey since its launch in September 2020. We have described the survey development, design, and domains in detail elsewhere [24,25].

### Measures

Participants were asked about their receipt of at least 1 dose of any of the approved COVID-19 vaccines in Canada and were also assessed for their level of mistrust in the COVID-19 vaccines.

Responses were rated on a 5-point scale ranging from 1 to 5, with 1 being “strongly disagree” and 5 being “strongly agree.” For the purpose of analyses, the responses were recoded, with those who responded “strongly disagree” or “disagree” coded as “trust” and those who responded “agree” and “strongly agree” coded as “mistrust.” Individuals who chose “neutral” were considered “undecided.”

We assessed sex, age, ethnicity, educational attainment, occupation, and household size based on self-reported data. Those who identified as First Nations, Inuit, Metis, Black (African or Caribbean), Filipino, Latin American or Hispanic, Southeast Asian (eg, Vietnamese, Cambodian, Malaysian, and Laotian), Arab, West Asian (eg, Iranian and Afghan), Korean, Japanese, and other prefer to self-describe, and prefer not to answer, were grouped under “other” due to their small sample sizes. Survey questions and response categories for the BC-Mix baseline and the follow-up survey are provided in Multimedia Appendices 1 and 2, respectively.

### Statistical Analyses

The domain on COVID-19 vaccine hesitancy and receipt was added to the survey on March 8, 2021. The analysis for this study was restricted to survey responses collected between March 8, 2021, and August 8, 2022. The survey data were weighted using 2016 Census data (Statistics Canada [26]) as the reference population. Employing Bethlehem’s weighting adjustment technique [27], age, sex, geography (Health Authority region), and ethnicity were used as auxiliary variables for weighting.

Participant characteristics were summarized using weighted frequencies and percentages, stratified by ethnicity. The distribution of mistrust in COVID-19 vaccines was examined for each ethnicity. We examined the distribution of vaccine receipt by mistrust status for each ethnicity. Logistic regression was used to examine the unadjusted and adjusted association between mistrust and vaccine receipt stratified by ethnicity. Confounders included ethnicity, sex, age, educational attainment, and household size while accounting for sampling design.

Data preparation, descriptive analyses, and all other analyses were performed in SAS software version 9.4 (SAS Institute Inc).

### Ethical Considerations

Informed consent was obtained from all participants. Ethical approval for this study was provided by the University of British Columbia Behavioral Research Ethics Board (H20-01785).

## Results

### Participant Profile

The analysis included 25,640 individuals. Overall, 55% (15,787/28,694) of the participants were male, and 41.7% (11,967/28,694) were older than 55 years. A total of 10.1% of participants identified as South Asian, 11% as Chinese, 62.7% as White, and 16.1% as of “other” ethnicity. Overall, 7.5% (2140/28,694) lived in a household size of >6, and this proportion was highest among those who identified as South Asian (17.2%, 499/2905) and “other” ethnicities (13.7%, 634/4641) compared with White (4.8%, 872/17,993) and Chinese (4.3%, 135/3156); see Table 1.

**Table 1:** Participant profile: comparison of characteristics by ethnicity (N=25,640).

Characteristic			South Asian, weighted n (%)		Chinese, weighted n (%)		White, weighted n (%)		Other, weighted n (%)		All, weighted n (%)
**Sex^a^**											
	Male	1504 (51.7)		1590 (50.4)		10,325 (57.4)		2369 (51.1)		15,787 (55)	
	Female	1402 (48.3)		1566 (49.6)		7667 (42.6)		2272 (48.9)		12,907 (45)	
**Age (years)^b^**											
	18-34	1012 (34.8)		709 (22.5)		3736 (20.8)		1615 (34.8)		7072 (24.6)	
	35-54	977 (33.6)		1162 (36.8)		5705 (31.7)		1812 (39)		9655 (33.6)	
	≥55	918 (31.6)		1285 (40.7)		8550 (47.5)		1214 (26.2)		11,967 (41.7)	
**Educational attainment^c^**											
	Below high school	59 (2)		58 (1.8)		370 (2.1)		181 (3.9)		669 (2.3)	
	Below bachelor	834 (28.7)		658 (20.9)		6910 (38.4)		1586 (34.2)		9988 (34.8)	
	University degree	973 (33.5)		1536 (48.7)		5436 (30.2)		1449 (31.2)		9394 (32.7)	
	Missing or unknown	1039 (35.8)		903 (28.6)		5276 (29.3)		1426 (30.7)		8644 (30.1)	
**Occupation^d^**											
	Essential workers	559 (19.2)		553 (17.5)		4199 (23.3)		1171 (25.2)		6482 (22.6)	
	Nonessential workers	638 (22)		903 (28.6)		4194 (23.3)		1003 (21.6)		6739 (23.5)	
	Do not work	401 (13.8)		551 (17.5)		3182 (17.7)		620 (13.4)		4755 (16.6)	
	Others	192 (6.6)		227 (7.2)		1166 (6.5)		338 (7.3)		1923 (6.7)	
	Prefer not to answer	173 (6)		62 (2)		293 (1.6)		212 (4.6)		740 (2.6)	
	Missing or unknown	942 (32.4)		860 (27.2)		4959 (27.6)		1297 (27.9)		8057 (28.1)	
**Household size^e^**											
	1	339 (11.7)		524 (16.6)		3258 (18.1)		681 (14.7)		4802 (16.7)	
	2	692 (23.8)		1226 (38.8)		8354 (46.4)		1431 (30.8)		11,702 (40.8)	
	3	473 (16.3)		593 (18.8)		2445 (13.6)		719 (15.5)		4231 (14.7)	
	4	634 (21.8)		515 (16.3)		2404 (13.4)		741 (16)		4293 (15)	
	5	224 (7.7)		163 (5.2)		612 (3.4)		389 (8.4)		1388 (4.8)	
	≥6	499 (17.2)		135 (4.3)		872 (4.8)		634 (13.7)		2140 (7.5)	
	Prefer not to answer	44 (1.5)		N/A^f^		48 (0.3)		46 (1)		138 (0.5)	

^a^South Asian: n=2906; Chinese: n=3156; White: n=17,992; Other: n=4641; All: n=28,694.

^b^South Asian: n=2907; Chinese: n=3156; White: n=17,991; Other: n=4641; All: n=28,694.

^c^South Asian: n=2905; Chinese: n=3155; White: n=17,992; Other: n=4642; All: n=28,695.

^d^South Asian: n=2905; Chinese: n=3156; White: n=17,993; Other: n=4641; All: n=28,696.

^e^South Asian: n=2905; Chinese: n=3156; White: n=17,993; Other: n=4641; All: n=28,694.

^f^N/A: not applicable.

### Vaccine Receipt by Ethnicity and Trust

Overall, 76.7% (22,010/28,696) of respondents reported having received at least 1 dose of the COVID-19 vaccines. Vaccine receipt was highest among individuals who identified as Chinese (2718/3156, 86.1%) followed by South Asian (2312/2905, 79.6%), White (13,583/17,991, 75.5%), and those belonging to the “other” ethnicity (3398/4642, 73.2%); see Table 2.

COVID-19 vaccine receipt by vaccine mistrust differed by ethnicity. Overall, 13.7% of the respondents reported mistrust of COVID-19 vaccines (Multimedia Appendix 3). There was a difference by ethnicity in the proportion of people who mistrusted the vaccine but still received it. This proportion was 50.6% among those who identified as South Asian, compared with 40.5% among Chinese, 26.9% among White, and 25.1% among individuals of “other” ethnicities (Table 3).

**Table 2:** Distribution of COVID-19 vaccine receipt and trust in COVID-19 vaccines by ethnicity in British Columbia, Canada.

Ethnicity	COVID-19 vaccine receipt, n/N (%)		Trust in COVID-19 vaccine, n/N (%)		
	Received	Did not receive	Mistrust	Trust	Undecided
South Asian	2312/2905 (79.6)	595/2917 (20.4)	239/2915 (8.2)	2276/2907 (78.3)	391/2896 (13.5)
White	13,583/17,991 (75.5)	4408/17,992 (24.5)	2769/17,981 (15.4)	13,499/17,999 (75)	1724/17,958 (9.6)
Chinese	2718/3156 (86.1)	439/3158 (13.9)	223/3141 (7.1)	2678/3154 (84.9)	255/3148 (8.1)
Other ethnicity	3398/4642 (73.2)	1243/4638 (26.8)	707/4651 (15.2)	3107/4637 (67)	826/4640 (17.8)
All	22,010/28,696 (76.7)	6684/28,687 (23.3)	3938/28,745 (13.7)	21,560/28,708 (75.1)	3197/28,802 (11.1)

**Table 3:** Characteristics of COVID-19 receipt by ethnicity in British Columbia.

Characteristic			South Asian										Chinese										White										Other Ethnicity										All			
			Received vaccine, n (%)			Did not receive vaccine, n (%)			Row^a^ (%)			Received vaccine, n (%)				Did not receive vaccine, n (%)			Row^a^ (%)			Received vaccine, n (%)				Did not receive vaccine, n (%)			Row^a^ (%)			Received vaccine, n (%)				Did not receive vaccine, n (%)			Row^a^ (%)			Received vaccine, n (%)				Did not receive vaccine, n (%)
**COVID-19 vaccine mistrust^b^**																																														
	Trust	1914 (82.8)		362 (60.9)			84.1			2404 (88.4)				275 (62.7)			89.7			11,710 (86.2)				1788 (40.6)			86.8			2720 (80.1)				387 (31.2)			87.5			18,748 (85.2)				2812 (42.1)		
	Mistrust	121 (5.2)		118 (19.9)			50.6			90 (3.3)				133 (30.3)			40.5			744 (5.5)				2025 (45.9)			26.9			178 (5.2)				530 (42.6)			25.1			1132 (5.1)				2805 (42)		
	Undecided	277 (12)		115 (19.3)			70.7			224 (8.2)				31 (7.1)			87.9			1129 (8.3)				595 (13.5)			65.5			500 (14.7)				326 (26.2)			60.5			2130 (9.7)				1067 (16)		
**Sex^c^**																																														
	Male	1136 (49.1)			368 (61.9)			75.5			1356 (49.9)				233 (53.2)			85.3			7421 (54.6)				2904 (65.9)			71.9			1618 (47.6)				751 (60.4)			68.3			11,531 (52.4)				4257 (63.7)	
	Female	1176 (50.9)			226 (38.1)			83.9			1361 (50.1)				205 (46.8)			86.9			6163 (45.4)				1504 (34.1)			80.4			1780 (52.4)				492 (39.6)			78.4			10,480 (47.6)				2427 (36.3)	
**Age (years)^d^**																																														
	18-34	725 (31.4)			287 (48.3)			71.7			569 (20.9)				140 (31.9)			80.3			2678 (19.7)				1058 (24)			71.7			1096 (32.3)				520 (41.8)			67.8			5067 (23)				2004 (30)	
	35-54	793 (34.3)			184 (30.9)			81.2			974 (35.8)				188 (42.9)			83.8			3965 (29.2)				1740 (39.5)			69.5			1329 (39.1)				482 (38.8)			73.4			7061 (32.1)				2594 (38.8)	
	≥55	794 (34.3)			124 (20.8)			86.5			1175 (43.2)				110 (25.2)			91.4			6941 (51.1)				1610 (36.5)			81.2			972 (28.6)				241 (19.4)			80.1			9882 (44.9)				2085 (31.2)	
**Educational attainment^e^**																																														
	Below high school	47 (2)			13 (2.1)			78.7			40 (1.5)				18 (4.2)			68.8			260 (1.9)				110 (2.5)			70.3			126 (3.7)				55 (4.4)			69.7			473 (2.1)				196 (2.9)	
	Below bachelor	675 (29.2)			159 (26.7)			81			583 (21.5)				75 (17.1)			88.6			5083 (37.4)				1827 (41.4)			73.6			1102 (32.4)				484 (38.9)			69.5			7443 (33.8)				2544 (38.1)	
	University degree	727 (31.4)			247 (41.5)			74.7			1337 (49.2)				200 (45.5)			87			4362 (32.1)				1073 (24.3)			80.3			1135 (33.4)				313 (25.2)			78.4			7561 (34.4)				1833 (27.4)	
	Missing/unknown	863 (37.3)			176 (29.6)			83.1			757 (27.9)				146 (33.2)			83.9			3878 (28.5)				1398 (31.7)			73.5			1035 (30.5)				391 (31.4)			72.6			6533 (29.7)				2111 (31.6)	
**Occupation^f^**																																														
	Essential workers	433 (18.7)			126 (21.2)			77.4			508 (18.7)				45 (10.3)			91.9			3025 (22.3)				1175 (26.6)			77.4			834 (24.6)				337 (27.1)			71.2			4799 (21.8)				1682 (25.2)	
	Nonessential workers	494 (21.4)			144 (24.2)			77.4			767 (28.2)				136 (31.1)			84.9			3292 (24.2)				902 (20.5)			78.5			763 (22.4)				240 (19.3)			76			5316 (24.2)				1423 (21.3)	
	Do not work	316 (13.7)			86 (14.4)			78.7			493 (18.1)				58 (13.3)			89.4			2568 (18.9)				613 (13.9)			80.7			449 (13.2)				172 (13.8)			72.3			3825 (17.4)				929 (13.9)	
	Others	138 (6)			54 (9.1)			71.9			165 (6.1)				61 (14)			73			847 (6.2)				319 (7.2)			71.9			261 (7.7)				77 (6.2)			77.2			1411 (6.4)				512 (7.7)	
	Prefer not to answer	143 (6.2)			31 (5.2)			82.3			57 (2.1)				5 (1.2)			91.7			183 (1.3)				110 (2.5)			62.5			138 (4.1)				74 (6)			65			521 (2.4)				220 (3.3)	
	Missing/unknown	788 (34.1)			154 (25.9)			83.7			728 (26.8)				132 (30.1)			84.6			3669 (27)				1289 (29.2)			74			954 (28.1)				343 (27.6)			73.5			6139 (27.9)				1918 (28.7)	
**Household size^g^**																																														
	1	289 (12.5)			51 (8.5)			85.1			454 (16.7)				70 (16)			86.6			2557 (18.8)				701 (15.9)			78.5			524 (15.4)				157 (12.7)			76.9			3823 (17.4)				979 (14.6)	
	2	543 (23.5)			149 (25.1)			78.4			1068 (39.3)				158 (36.1)			87.1			6570 (48.4)				1784 (40.5)			78.6			1107 (32.6)				323 (26)			77.4			9287 (42.2)				2415 (36.1)	
	3	390 (16.9)			83 (14)			82.4			513 (18.9)				80 (18.2)			86.6			1786 (13.1)				659 (15)			73			535 (15.8)				184 (14.8)			74.4			3224 (14.6)				1006 (15.1)	
	4	506 (21.9)			128 (21.5)			79.8			465 (17.1)				51 (11.5)			90.2			1733 (12.8)				671 (15.2)			72.1			547 (16.1)				194 (15.6)			73.8			3250 (14.8)				1043 (15.6)	
	5	198 (8.6)			26 (4.3)			88.5			120 (4.4)				44 (9.9)			73.3			402 (3)				210 (4.8)			65.7			260 (7.7)				129 (10.4)			66.9			980 (4.5)				408 (6.1)	
	≥6	353 (15.2)			147 (24.7)			70.6			99 (3.6)				36 (8.3)			73.1			516 (3.8)				356 (8.1)			59.1			397 (11.7)				237 (19.1)			59.1			1364 (6.2)				776 (11.6)	
	Prefer not to answer	34 (1.5)			11 (1.8)			75.8			0				0			0			21 (0.2)				27 (0.6)			43			27 (0.8)				19 (1.5)			59.5			82 (0.4)				57 (0.8)	

^a^Vaccine received variable (proportion of people who received the vaccine among those who trusted it, mistrusted it, or were undecided).

^b^South Asian: received vaccine: n=2312, did not receive: n=595; Chinese: received vaccine: n=2718, did not receive: n=439; White: received vaccine: n=13,583, did not receive: n=4408; Other: received vaccine: n=3398, did not receive: n=1243; All: received vaccine: n=22,010, did not receive: n=6684.

^c^South Asian: received vaccine: n=2312, did not receive: n=594; Chinese: received vaccine: n=2717, did not receive: n=438; White: received vaccine: n=13,584, did not receive: n=4408; Other: received vaccine: n=3398, did not receive: n=1243; All: received vaccine: n=22,011, did not receive: n=6684.

^d^South Asian: received vaccine: n=2312, did not receive: n=595; Chinese: received vaccine: n=2718, did not receive: n=438; White: received vaccine: n=13,584, did not receive: n=4408; Other: received vaccine: n=3397, did not receive: n=1243; All: received vaccine: n=22,010, did not receive: n=6683.

^e^South Asian: received vaccine: n=2312, did not receive: n=595; Chinese: received vaccine: n=2717, did not receive: n=439; White: received vaccine: n=13,583, did not receive: n=4408; Other: received vaccine: n=3398, did not receive: n=1243; All: received vaccine: n=22,010, did not receive: n=6684.

^f^South Asian: received vaccine: n=2312, did not receive: n=595; Chinese: received vaccine: n=2718, did not receive: n=437; White: received vaccine: n=13,584, did not receive: n=4408; Other: received vaccine: n=3399, did not receive: n=1243; All: received vaccine: n=22,011, did not receive: n=6684.

^g^South Asian: received vaccine: n=2313, did not receive: n=595; Chinese: received vaccine: n=2719, did not receive: n=439; White: received vaccine: n=13,585, did not receive: n=4408; Other: received vaccine: n=3397, did not receive: n=1243; All: received vaccine: n=22,010, did not receive: n=6684.

### Association Between Mistrust and Vaccine Receipt

In the unadjusted logistic regression model, those who identified as Chinese were significantly more likely to have received the vaccine (odds ratio [OR] 2.01, 95% CI 1.55-2.62) compared with those who identified as White. Although those who identified as South Asians were also more likely to have received the vaccine, this effect was not significant. Individuals who mistrusted COVID-19 vaccines had 94% reduced odds of vaccine receipt (OR 0.06, 95% CI 0.05-0.07) compared with those who trusted vaccines. In the models stratified by ethnicity, mistrust was associated with 81% (OR 0.19, 95% CI 0.09-0.43), 92% (OR 0.08, 95% CI 0.03-0.18), 94% (OR 0.06, 95% CI 0.05-0.07), and 95% (OR 0.05, 95% CI 0.03-0.07) reduced odds of vaccine receipt among those who identified as South Asian, Chinese, White, and those of other ethnicities, respectively. Individuals who were undecided or neutral regarding whether they trusted or mistrusted the vaccine had 70% reduced odds of receiving the vaccine. Among undecided South Asians, reduced odds of receiving the vaccine were 54% (OR 0.46, 95% CI 0.22-0.94) compared with 71% in the White (OR 0.29, 95% CI 0.24-0.34) and 78% in the “other” (OR 0.22, 95% CI 0.16-0.30). In those identifying as Chinese, the reduction in odds was not significant (Table 4).

After adjusting for age, sex, educational attainment, and household size, mistrust was associated with a 93% reduced odds of vaccine receipt (adjusted OR [aOR] 0.07, 95% CI 0.06-0.08). There was some attenuation of the effect of ethnicity, but results were similar for those who identified as Chinese (aOR 1.63, 95% CI 1.20-2.20), those who identified as South Asian (aOR 1.08, 95% CI 0.93-1.25), and those of “other” ethnicities (aOR 1.17, 95% CI 0.86-1.61).

The adjusted magnitude of association between mistrust and vaccine receipt was greatest among individuals of “other” ethnicities (aOR 0.05, 95% CI 0.03-0.07), followed by those who identified as White (aOR 0.06, 95% CI 0.05-0.07), those who identified as Chinese (aOR 0.08, 95% CI 0.03-0.20), and those who identified as South Asian (aOR 0.17, 95% CI 0.07-0.38). A significant association was observed between those who were undecided and those who received the vaccine among individuals identifying as White (OR 0.30, 95% CI 0.25-0.36) and of “other” ethnicity (OR 0.22, 95% CI 0.16-0.31; Table 5).

Other covariates associated with vaccine receipt included female sex (OR 1.59, 95% CI 1.45-1.75), age >55 years compared with 18-34 years (OR 1.88, 95% CI 1.64-2.15), and a university education compared with below high school education (OR 1.71, 95% CI 1.22-2.38; Table 5).

**Table 4:** Unadjusted association between mistrust and vaccine receipt by ethnicity in British Columbia.

Characteristic		Unadjusted odds ratio (95% CI)				
		South Asian	Chinese	White	Other ethnicity	All
**Ethnicity**						
	White	N/A^a^	N/A	N/A	N/A	Reference
	Chinese	N/A	N/A	N/A	N/A	2.01 (1.55-2.62)
	Other ethnicity	N/A	N/A	N/A	N/A	0.89 (0.77-1.02)
	South Asian	N/A	N/A	N/A	N/A	1.26 (0.97-1.65)
**Mistrust in vaccines**						
	Yes	0.19 (0.09-0.43)	0.08 (0.03-0.18)	0.06 (0.05-0.07)	0.05 (0.03-0.07)	0.06 (0.05-0.07)
	No	Reference	Reference	Reference	Reference	Reference
	Undecided	0.46 (0.22-0.94)	0.83 (0.33-2.05)	0.29 (0.24-0.34)	0.22 (0.16-0.30)	0.30 (0.26-0.35)
**Sex**						
	Male	Reference	Reference	Reference	Reference	Reference
	Female	1.68 (1.03-2.75)	1.14 (0.69-1.90)	1.60 (1.47-1.75)	1.68 (1.33-2.13)	1.59 (1.45-1.75)
**Age (years)**						
	18-34	Reference	Reference	Reference	Reference	Reference
	35-54	1.71 (0.90-3.23)	1.27 (0.67-2.43)	0.90 (0.76-1.07)	1.31 (0.96-1.78)	1.08 (0.93-1.25)
	≥55	2.54 (1.30-4.96)	2.62 (1.29-5.30)	1.70 (1.46-1.99)	1.91 (1.39-2.63)	1.88 (1.64-2.15)
**Educational attainment**						
	Below high school	Reference	Reference	Reference	Reference	Reference
	Below bachelor	1.15 (0.19-6.95)	3.53 (0.68-18.39)	1.18 (0.83-1.67)	0.99 (0.51-1.93)	1.21 (0.87-1.68)
	University degree	0.80 (0.14-4.62)	3.04 (0.63-14.76)	1.72 (1.20-2.45)	1.58 (0.81-3.06)	1.71 (1.22-2.38)
	Missing/unknown	1.33 (0.23-7.66)	2.36 (0.47-11.80)	1.17 (0.82-1.67)	1.15 (0.60-2.22)	1.28 (0.92-1.78)
**Household size**						
	1	Reference	Reference	Reference	Reference	Reference
	2	0.64 (0.22-1.82)	1.05 (0.50-2.21)	1.01 (0.88-1.16)	1.03 (0.71-1.49)	0.99 (0.86-1.13)
	3	0.82 (0.25-2.71)	1.00 (0.42-2.39)	0.74 (0.62-0.90)	0.87 (0.57-1.34)	0.82 (0.68-0.99)
	4	0.69 (0.23-2.08)	1.43 (0.59-3.44)	0.71 (0.59-0.85)	0.85 (0.55-1.30)	0.80 (0.67-0.96)
	5	1.35 (0.33-5.57)	0.43 (0.13-1.37)	0.53 (0.39-0.70)	0.61 (0.35-1.07)	0.62 (0.47-0.81)
	≥6	0.42 (0.14-1.27)	0.42 (0.14-1.32)	0.40 (0.31-0.51)	0.50 (0.31-0.83)	0.45 (0.36-0.57)
	Prefer not to answer	0.55 (0.05-6.26)	N/A	0.21 (0.08-0.522)	0.44 (0.12-1.64)	0.37 (0.17-0.82)

^a^N/A: not applicable.

**Table 5:** Adjusted association between mistrust and vaccine receipt by ethnicity in British Columbia.

Characteristic			Adjusted odds ratio (95% CI)								
			South Asian		Chinese		White		Other ethnicity		All
**Ethnicity**											
	White	N/A^a^		N/A		N/A		N/A		Reference	
	Chinese	N/A		N/A		N/A		N/A		1.63 (1.20-2.20)	
	Other ethnicity	N/A		N/A		N/A		N/A		1.08 (0.93-1.25)	
	South Asian	N/A		N/A		N/A		N/A		1.17 (0.86-1.61)	
**Mistrust in vaccines**											
	Yes	0.17 (0.07-0.38)		0.08 (0.03-0.20)		0.06 (0.05-0.07)		0.05 (0.03-0.07)		0.07 (0.06-0.08)	
	No	Reference		Reference		Reference		Reference		Reference	
	Undecided	0.47 (0.21-1.07)		0. 81 (0.31-2.11)		0.30 (0.25-0.36)		0.22 (0.16-0.31)		0.32 (0.27-0.37)	
**Sex**											
	Male	Reference		Reference		Reference		Reference		Reference	
	Female	1.79 (1.05-3.06)		0.84 (0.46-1.54)		1.14 (1.03-1.27)		1.39 (1.07-1.81)		1.24 (1.11-1.38)	
**Age**											
	18-34	Reference		Reference		Reference		Reference		Reference	
	35-54	1.97 (0.97-3.99)		1.36 (0.64-2.87)		1.03 (0.84-1.26)		1.10 (0.78-1.55)		1.18 (1.00-1.40)	
	≥55	2.78 (1.42-5.46)		2.16 (0.97-4.82)		1.54 (1.28-1.86)		1.75 (1.23-2.50)		1.78 (1.52-2.10)	
**Educational attainment**											
	Below high school	Reference		Reference		Reference		Reference		Reference	
	Below bachelor	0.83 (0.11-6.36)		2.70 (0.68-10.81)		0.96 (0.62-1.50)		0.74 (0.31-2.06)		0.98 (0.65-1.48)	
	University degree	0.57 (0.08-4.26)		2.20 (0.61-7.86)		1.07 (0.68-1.66)		0.91 (0.65-1.50)		1.04 (0.69-1.57)	
	Missing/unknown	1.07 (0.14-7.98)		1.83 (0.51-6.52)		1.26 (0.81-1.97)		1.04 (0.40-2.71)		1.24 (0.82-1.87)	
**Household size**											
	1	Reference		Reference		Reference		Reference		Reference	
	2	0.51 (0.18-1.46)		1.03 (0.48-2.25)		0.88 (0.75-1.03)		0.98 (0.65-1.50)		0.88 (0.75-1.03)	
	3	0.77 (0.23-2.58)		1.10 (0.42-2.88)		0.85 (0.69-1.08)		1.36 (0.81-2.27)		0.94 (0.76-1.16)	
	4	0.61 (0.20 (1.87)		1.50 (0.59-3.82)		0.86 (0.69-1.08)		1.07 (0.65-1.77)		0.91 (0.74-1.12)	
	5	1.21 (0.27-5.47)		0.43 (0.13-1.36)		0.84 (0.61-1.16)		0.85 (0.43-1.68)		0.81 (0.58-1.12)	
	≥6	0.45 (0.15-1.39)		0.70 (0.26-1.88)		0.62 (0.46-0.83)		1.03 (0.60-1.76)		0.69 (0.53-0.89)	
	Prefer not to answer	N/A		N/A		N/A		N/A		0.66 (0.26-1.70)	

^a^N/A: not applicable.

## Discussion

This study assessed the role of self-reported mistrust of COVID-19 vaccines in vaccine receipt among different ethnic groups in BC, Canada. We found that mistrust was lower among individuals who identified as Chinese and South Asian. Vaccine uptake was higher among people identifying as Chinese compared with people identifying as White. Among those who identified as South Asian, vaccine receipt was higher than the White population, but this difference was not statistically significant. Mistrust of the vaccine was significantly associated with a reduced odds of vaccine receipt, regardless of race and ethnicity. However, the magnitude of association between mistrust and vaccine receipt differed by ethnicity, with vaccine trust playing the least important role in vaccine receipt among those who identified as South Asian. Only 8.2% of those who identified as South Asian reported mistrust (Multimedia Appendix 3), and despite that, vaccine receipt among this group was still the highest (50.6%) compared with 40.5% among those who identified as Chinese, 26.9% among White, and 25.1% among individuals of “other” ethnicities. Indecisiveness about whether the vaccine was to be trusted or not also had a significant association with vaccine receipt. In the “undecided” group, those who identified as Chinese and South Asians had the highest proportion of vaccine receipt (88% and 71%, respectively) compared with White (66%) and those identifying as of “other” ethnicities (61%).

Our results contrast with findings from other studies that have reported a higher prevalence of vaccine hesitancy and lower vaccine receipt in racialized groups. For instance, although the United Kingdom became the first country to approve COVID-19 vaccines for emergency use in December 2020 and took the lead in vaccinating its population, it faced elevated vaccine hesitancy among Black, South Asian, and other ethnic minorities [9,28-31]. Similarly, US-based studies reported greater vaccine hesitancy and lower vaccine receipt among African Americans and Hispanics compared with the White American population [32-34].

Several reasons have been cited for this widespread hesitancy and lower vaccine receipt among ethnic groups. Unknown future effects, side effects of vaccines, a lack of trust in government and pharmaceutical industry, and historical inequities and racism have been implicated in vaccine hesitancy among people of color in both the United Kingdom and United States [9,32,34,35]. Contrary to this, overall vaccine acceptance among people designated as a visible minority in Canada has been relatively high with 74.8% reporting being “very” or “somewhat willing” to receive COVID-19 vaccines [2]. Of the 11 distinct ethnic groups surveyed in Canada, the South Asian group had one of the highest proportions of vaccine acceptance (82.5%) third in line to Japanese at 87.6% and Koreans at 85.5% compared with 77.7% in those who were not a visible minority [2].

In our study, mistrust of the vaccine as well as indecisiveness whether to trust the vaccine or not was significantly associated with a reduced odds of vaccine receipt regardless of race and ethnicity. The timing of our survey may explain some of these trends. The BC-Mix survey was launched in September 2020, with the vaccine questionnaire being added in March 2021—approximately 3 months after nationwide vaccination in Canada began. Limited information about the novel SARS-CoV-2 virus and concerns over safety and efficacy of a vaccine developed in a very short time may have generated a considerable number of people indicating reluctance to get vaccinated as reported in studies from the United States and France [36-38]. This may, to some degree, also explain a high prevalence of indecisiveness in our study, which, as our results demonstrate, had a significant association with willingness to get vaccinated. Dearth of accurate information, misinformation or mixed messages coupled with lack of culturally tailored, and targeted messaging in the early months of the pandemic may have further fueled skepticism or indecisiveness among various population groups—specifically the already racialized and marginalized communities. However, despite this, mistrust and indecisiveness appeared to have less of an impact on vaccine receipt among those who identified as Chinese (88%) and South Asian (70%).

There may be several potential explanatory factors for why people who identified as South Asian received a vaccine despite reporting vaccine mistrust. First, the decision to get vaccinated despite mistrust may be due to targeted interventions by BC Health Authorities to increase vaccine uptake within the South Asian community. These interventions may have, to some degree, overridden the initial fears and mistrust at the individual level. Second, a higher risk perception of COVID-19 severe outcomes, perhaps from having witnessed the high burden of disease and mortality among community members in both BC and Ontario [39] coupled with messaging from primary care physicians and community-based health workers framing vaccines as a “lifesaver,” may have driven many to opt for the vaccine despite their initial mistrust [40]. Third, subjective norms could be another explanation whereby members of the South Asian community, mistrustful of the vaccines, felt pressured to “give in” as other members of the family or one’s social circle appeared to be strongly supportive of vaccination [40]. Fourth, South Asian grandparents are 8 times more likely to live with their grandchildren (National Household Survey 2011) [41], and this seems to be reflected in our sample, where 17.2% of South Asian individuals (highest compared with any other ethnic group) lived in a household size of 6 or greater. Fear of exposing the older adult in the family to COVID-19 may have been another factor pushing those who were mistrustful of the vaccine to get vaccinated. Lastly, fear of consequences for not following government regulations, such as those related to international travel or accessing public facilities, may have convinced some to get vaccinated despite mistrust [40]. Future research should focus on some of these psychosocial and behavioral factors that could explain greater acceptance and receipt of COVID-19 vaccines among the South Asian population.

Contrasting findings have been observed in UK and US studies. In the UK Household Longitudinal Study, higher vaccine hesitancy (among those who had not yet received a vaccine) was seen in most minority ethnic groups compared with the White British or Irish group. The highest odds were seen in the Black or Black British group (OR 13.42, 95% CI 6.86-26.24) and the Pakistani and Bangladeshi South Asian subgroups (OR 2.54, 95% CI 1.19-5.44), and adjustment for covariates made relatively little difference to these associations [29]. In the United States, Wu et al [35] examined the mechanisms behind COVID-19 vaccine acceptance in 3 Asian American ethnic groups (East Asian, Southeast Asian, and South Asian), including how sociodemographic characteristics and racism predicted COVID-19 vaccine perceptions. Trust worthiness of public health agency’s recommendation for COVID-19 vaccines was not associated with ethnicity. When predicting COVID-19 vaccine safety concerns, the South Asian group (compared with non-Asian) reported significantly higher safety concerns, which was mediated by racism [35].

Despite some methodological differences, contrasting findings of vaccine hesitancy and receipt among the South Asian population in Canada compared with the United Kingdom raises thought-provoking questions as to why the Canadian South Asian population might be more accepting and trusting of COVID-19 vaccines than their British counterparts. This finding may partly be explained by subgroup differences that exist among South Asian diaspora. It is important to note that South Asians (people originating from or having ancestry in India, Pakistan, Bangladesh, Nepal, Sri Lanka, Maldives, and Bhutan) are not a monolithic group but have considerable heterogeneity based on country of origin, religion, culture, and language. In the United Kingdom, while Indians comprise the biggest South Asian subgroup (similar to Canada), people of Pakistani and Bangladeshi origin also have a significant presence. In fact, when put together, the group comprising people with Pakistani and Bangladeshi origins surpass Indians in numbers [42]. Significant differences in health behaviors and health outcomes for chronic diseases have been reported among various South Asian subgroups based on religion and country of origin [43]. Research exploring vaccine receipt and hesitancy among various subgroups of ethnic denominations in the United Kingdom reveals greater hesitancy and mistrust among the South Asian population originating from predominantly Muslim countries such as Bangladesh and Pakistan compared with India [29,44].

In the province of BC, Indians, and specifically Indians of Punjabi origin who are settled in the province of Punjab and identify predominantly with Sikh religion, constitute the largest South Asian subgroup, whereas the number of Pakistanis and, especially, Bangladeshis is relatively small [45]. Although we do not have a subgroup breakdown of the South Asian sample, it is possible that our study sample predominantly constitutes South Asian individuals originating from India, and thus our findings may be reflective of the subgroup differences highlighted by UK-based studies where South Asians of Indian origin are more accepting of the vaccine than Pakistanis or Bangladeshis [29,44]. Vaccine acceptance has been a challenge in many Muslim countries where rejection of immunization is widely prevalent on the grounds that the vaccine contains animal byproducts that are not *halal* (kosher) or unfounded claims that it may cause infertility [46-48]. Subgroup differences based on country of origin and religion require further investigations to understand vaccine receipt and hesitancy among the South Asian population, which may inform effective vaccine receipt strategies for COVID-19 and other vaccines in future.

Another plausible explanation for the difference in vaccination receipt between the South Asian population in the United Kingdom compared with Canada could be greater anti-Muslim sentiment and social exclusion experienced by the Muslim community in the United Kingdom [49]. This may therefore result in greater mistrust between political leadership and Muslims. The South Asian population in the United Kingdom, specifically Muslims, have been targets of increased hate crimes associated with the war on terror after the events of September 11, 2001, and high-profile police action after the 2005 London bombings, which resulted in alienation of the Muslim community and greater distrust of the government [50]. Although anti-Muslim sentiment in Canada has also increased, a 2016 national survey examining the relationship between Canadian Muslims and Canadian society at large revealed that Muslims, nearly 7 in 10 of whom are immigrants, were generally happy to be in Canada and 84% of Muslims surveyed believed that they were treated better in Canada than their coreligionists in other Western countries, a number that has increased since 2006 [51]. The majority also expressed optimism regarding their relationship with the government. In contrast, a 2016 British examination about the state of Muslim citizens’ opinions on a broad range of topics revealed that relations between Britain’s political establishment and the leadership of the Muslim community are at a nadir [50]. Higher vaccine acceptance among the South Asian population in Canada may thus partly be explained by a higher degree of social inclusion experienced by one of the major subgroups (ie, Muslims) within the South Asian diaspora compared with Muslims in the United Kingdom. This however is speculative and needs to be investigated in future research as an understanding of the contexts and fluidities underlying the knowledge and beliefs and lived experiences about health and immunization in diverse communities is critical for designing effective interventions [52].

Higher vaccine receipt among the South Asian population in Canada could also be related to potential differences in vaccine program administration. For instance, ethnic minorities in the United Kingdom were more likely to receive an influenza vaccine administered by general practitioners in communities with whom they may have a trusting relationship, while COVID-19 vaccines were administered through mass vaccination centers or hospital hub [53]. Moreover, residential segregation driven by systemic racism may have resulted in additional barriers in terms of travel time and transport-related costs to accessing centralized vaccination sites for some communities in the United Kingdom [53]. Although a somewhat similar vaccination setup was rolled out in BC, working at a grassroots level to get the populations most impacted by the pandemic onboard may explain lower resistance and higher levels of vaccine receipt among these groups. For instance, in BC, the greatest concentration of the South Asian population is in the Fraser Health region where they make up almost 15% of the population [54]. The South Asian Health Institute was established in 2013 to improve health for South Asian people and reduce chronic disease burden. This institute works closely with Fraser Health Authority (a regional health authority), community leaders and stakeholders to promote health and well-being and has ongoing programs targeting lifestyle behaviors like diet, physical activity as well as mental health, and drug toxicity. During the pandemic, South Asian Health Institute and several South Asian community volunteer groups and clinic partners were able to leverage their prepandemic established relationships and networks within the South Asian community and were at the forefront in reaching out to the community via “in-reach” clinics set up in community centers and places of worship [55]. These clinics were staffed with South Asian team members providing support for registration with the provincial COVID-19 vaccination program. Moreover, translation services for COVID-19 information were available in 145 languages including Hindi, Urdu, Punjabi, Tamil, and Chinese [55]. These measures removed many barriers around language, transportation, technological issues, health system navigation, and vaccine hesitancy. This may partly explain high vaccine receipt even among groups that were mistrustful of the vaccine.

### Limitations

This study has some limitations. Because this was a web-based survey in English, it may have excluded many South Asians and Chinese with limited digital literacy and fluency in English. Thus, the findings cannot be generalized to these population groups. The findings of our survey may be subject to social desirability bias; given the self-reported nature of the data, it is possible that participants responded in ways that they deemed socially acceptable. We were also unable to provide further disaggregation of ethnicity to examine differences among certain ethnic groups and subgroups (eg, Indian, Pakistani, and Bangladeshi groups in the South Asian diaspora). Additionally, we did not investigate the source or cause of mistrust that limits our ability to identify modifiable factors for future vaccination strategies. Despite these limitations, this is one of the largest surveys examining vaccine receipt and mistrust among South Asians in Canada.

### Conclusions

Factors that influence vaccine acceptance are multifaceted and often, as research shows, shaped by complex, sociocultural, historical, institutional, clinical, community, and governmental levels [56]. In contrast to findings among South Asians in the United Kingdom, our study found higher levels of vaccine acceptance, and similar levels of vaccine uptake, compared with individuals identifying as White. We also found high vaccine receipt among those South Asians who mistrusted the vaccine. The contrasting results may have a range of explanations—including important differences between the South Asian populations in the United Kingdom and Canada in terms of country of origin, religion, racism, social exclusion, alienation, and consequent mistrust in the government and systems—all of which have an impact on vaccination uptake. Future research needs to focus on disentangling the potential influence of these factors on vaccine receipt. An understanding of factors that facilitate vaccine receipt among the South Asian population residing in Canada will enable researchers and health professionals to tailor this information and use it for developing targeted interventions and informed national vaccination programs for South Asians residing in regions with elevated vaccine hesitancy.

## Appendix

Multimedia Appendix 1 BC COVID-19 population mixing patterns baseline survey (BC-Mix).

Multimedia Appendix 2 BC COVID-19 population mixing patterns follow-up survey (BC-Mix).

Multimedia Appendix 3 Mistrust in COVID-19 vaccine by ethnicity in British Columbia.

## Abbreviations

aOR: adjusted odds ratio
BC: British Columbia
OR: odds ratio

## Data Availability

The data sets generated during and/or analyzed during this study are available from the corresponding author on reasonable request.
