# Supplementary material for: Ethnic Disparities in COVID-19 Vaccine Mistrust and Receipt in British Columbia, Canada: Population Survey
Source: JMIR Public Health Surveill. 2024 Feb 16;10:e48466. doi: 10.2196/48466 (PMC10896316; doi:10.2196/48466)
Supplement: Multimedia Appendix 1 [file publichealth_v10i1e48466_app1.pdf]

BCMIX- COVID-19 SURVEY

Introduction & Informed Consent

COVID-19- BC Population Mixing Patterns Survey

Introduction

Thank you for considering completing this survey. This survey has been developed as a result of the continuing global spread of COVID-19. To monitor changes in COVID-19 transmission, the BC Centre for Disease Control ("BCCDC") is developing early warning systems to warn when COVID-19 transmission might increase. To develop these systems, we will use surveys and mobility data to assess changes in physical distancing behaviours and the impact of those changes on COVID-19 transmission.

The survey should take 10-15 minutes to complete. At the end of the survey, we will ask for your permission to link your survey responses to other healthcare administration databases to help us in performing meaningful population level analyses.

Responses from the survey will be used to inform provincial COVID-19 plans going forward. To compensate for your time, you will be entered into a draw to receive 1 of 200 gift cards worth $25.

Before you begin the survey, please click on the link below to download and read the consent form:

Consent Form BCMix Survey v4

Do you consent to participate in survey?

- ○ Yes
- ○ No

Participant Demographic Information

What is your age?

- ○ 18-24
- ○ 25-34
- ○ 35-44
- ○ 45-54
- ○ 55-64
- ○ 65-74
- ○ 75 or greater
- ○ Prefer not to answer

What is your sex?

- ○ Male
- ○ Female
- ○ Other
- ○ Prefer not to answer

Do you consider yourself to be (check all that apply)

- ☐ First Nations
- ☐ Métis
- ☐ Inuit
- ☐ White (European descent)
- ☐ Chinese
- ☐ South Asian (e.g. East Indian, Pakistani, Sri Lankan)
- ☐ Black (e.g. African or Caribbean)
- ☐ Filipino
- ☐ Latin American/ Hispanic

- ☐ Southeast Asian (e.g. Vietnamese, Cambodian, Malaysian, Laotian)
- ☐ Arab
- ☐ West Asian (e.g. Iranian, Afghan)
- ☐ Korean
- ☐ Japanese
- ☐ ______ Other, prefer to self describe
- ☐ Prefer not to answer

Perception of COVID-19 Response

How satisfied are you with how COVID-19 has been managed in the province?

- ○ ______ Very satisfied (you may include reasons below)
- ○ ______ Satisfied (you may include reasons below)
- ○ ______ Not sure (you may include reasons below)
- ○ ______ Dissatisfied (you may include reasons below)
- ○ ______ Very dissatisfied (you may include reasons below)
- ○ Prefer not to answer

Household Characteristics

How many **adults** live in your household? If you live alone, choose 1. If you live with one person choose 2 etc.

By household, we mean anyone living at the same address as you, that you share a kitchen with.

- ○ 1
- ○ 2

- 3
- 4
- 5
- 6 or more
- Prefer not to answer

How many **children (under 18 years)** live in your household?

By household, we mean anyone living at the same address as you, that you share a kitchen with.

- 0
- 1
- 2
- 3
- 4
- 5
- 6 or more
- Prefer not to answer

Vaccination

Have you received the COVID-19 vaccine?

- Yes
- No

When did you receive your 1st COVID-19 vaccine shot?

	Month	Day	Year

Please Select:			

Have you received your 2nd COVID-19 vaccine shot?

- ○ Yes
- ○ No
- ○ I don't know/Can't remember

When did you receive your 2nd COVID-19 vaccine shot?

	Month	Day	Year
Please Select:			

Have you received your 3rd COVID-19 vaccine shot?

- ○ Yes
- ○ No
- ○ I don't know/Can't remember

When did you receive your 3rd COVID-19 vaccine shot?

	Month	Day	Year
Please Select:			

Have you received your 4th COVID-19 vaccine shot?

- ○ Yes
- ○ No
- ○ I don't know/Can't remember

When did you receive your 4th COVID-19 vaccine shot?

	Month	Day	Year
Please Select:			

Have you received your 5th COVID-19 vaccine shot?

- ○ Yes
- ○ No
- ○ I don't know/Can't remember

When did you receive your 5th COVID-19 vaccine shot?

	Month	Day	Year
Please Select:			

Indicate your level of agreement with the following statements.

I believe I am at risk of becoming infected with COVID-19.

- ○ 1-Strongly Disagree
- ○ 2-Disagree
- ○ 3-Neutral
- ○ 4-Agree
- ○ 5-Strongly Agree

With the way my life is, I believe I am at a high risk of getting COVID-19 (e.g.

risks at my work, recreational activities, people I live with, etc.)

- 1-Strongly Disagree
- 2-Disagree
- 3-Neutral
- 4-Agree
- 5-Strongly Agree

I believe a COVID-19 Vaccine will protect me from getting the virus.

- 1-Strongly Disagree
- 2-Disagree
- 3-Neutral
- 4-Agree
- 5-Strongly Agree

I believe a COVID-19 vaccine will decrease my chance of getting seriously ill from COVID-19.

- 1-Strongly Disagree
- 2-Disagree
- 3-Neutral
- 4-Agree
- 5-Strongly Agree

I ***do not*** trust the COVID-19 vaccine.

- 1-Strongly Disagree
- 2-Disagree
- 3-Neutral
- 4-Agree

- 5-Strongly Agree

I am concerned about the effectiveness of the COVID-19 vaccination.

- 1-Strongly Disagree
- 2-Disagree
- 3-Neutral
- 4-Agree
- 5-Strongly Agree

I am concerned about the safety of the COVID-19 vaccination.

- 1-Strongly Disagree
- 2-Disagree
- 3-Neutral
- 4-Agree
- 5-Strongly Agree

Most of the people I know are getting or have received the COVID-19 vaccine.

- 1-Strongly Disagree
- 2-Disagree
- 3- Don't know
- 4-Agree
- 5-Strongly Agree

Most of the people who are important to me (my family, relatives and/or friends) think I should get the COVID-19 vaccine.

- 1-Strongly Disagree

- 2-Disagree
- 3-Don't know
- 4-Agree
- 5-Strongly Agree

If I choose to get the COVID-19 vaccine, I believe it will be easy to get it.

- 1-Strongly Disagree
- 2-Disagree
- 3-Don't know
- 4-Agree
- 5-Strongly Agree

I plan to get the COVID-19 vaccine.

- 1-Strongly Disagree
- 2-Disagree
- 3-Undecided
- 4-Agree
- 5-Strongly Agree

COVID-19 Diagnosis

Since January 2020, have you had any of the following symptoms? Check all that apply

- [ ] I have not had any symptoms
- [ ] Headache
- [ ] Fever
- [ ] Stuffy nose/ congestion

- [ ] Loss of smell or taste
- [ ] New or worsening cough
- [ ] Difficulty breathing/shortness of breath
- [ ] Confusion
- [ ] Vomiting
- [ ] Chills
- [ ] Weakness
- [ ] Muscle pain
- [ ] Fatigue
- [ ] Nausea
- [ ] Diarrhea
- [ ] ________ Other; please specify
- [ ] I don't know
- [ ] Prefer not to answer

When did your first symptom start? If you do not remember, enter your best guess.

	Month	Day	Year
Please select:			

Have you done any of the following for these symptoms? (please check all that apply)

- [ ] Called 811
- [ ] Called Family doctor/ GP
- [ ] Consulted family doc through telehealth
- [ ] Visited, Family doctor's /GP office
- [ ] Visited, Community/public health clinic
- [ ] Visited, hospital emergency department
- [ ] Visited, urgent care clinic

- [ ] Visited, COVID testing centre
- [ ] Been admitted to hospital
- [ ] Used home remedies
- [ ] Treated symptoms with over the counter medications (Tylenol, etc.)
- [ ] ______ Others; please specify
- [ ] None of the above
- [ ] Prefer not to answer

Before these symptoms, had you been in close contact with anyone who **either**: **(A)** had any of those symptoms [fever, new or worsening cough, headache, chills, weakness, muscle pain, stuffy nose/congestion, sore throat, difficulty breathing/shortness of breath, nausea, diarrhea, fatigue, loss of smell or taste, confusion, vomiting]; **OR (B)** was diagnosed positive for COVID-19 within 14 days before you felt sick?

- ○ Yes
- ○ No
- ○ I don't know
- ○ Prefer not to answer

Did you isolate, or stay away from your workplace or educational facility?

- ○ Yes
- ○ No
- ○ I don't know
- ○ Prefer not to answer

Have you been tested for COVID-19?

- ○ Yes

- No
- Prefer not to answer

Did you test positive for COVID-19?

- Yes
- No
- I don't know/Prefer not to answer

When was your most recent COVID-19 test? If you do not remember, enter your best guess.

	Month	Day	Year
Please select:			

In your most recent COVID-19 test, what type of COVID-19 test did you take?

- PCR test
- Rapid Antigen Test/Lateral Flow Test
- I don't know/Prefer not to answer

Why were you tested?

- I was symptomatic
- I was exposed to someone who tested positive for COVID-19 but currently asymptomatic
- I needed it for travel-related reasons
- Other reasons
- I don't know/Prefer not to answer

Has anyone in your household **either**: **(A)** had any of the following symptoms: fever, new or worsening cough, headache, chills, weakness, muscle pain, stuffy nose/congestion, sore throat, difficulty breathing/shortness of breath, nausea, diarrhea, fatigue, loss of smell or taste, confusion, vomiting; **OR (B)** tested positive for COVID-19 since January 2020?

- Yes
- No
- I don't know
- Prefer not to answer

When did **their** first symptom start? If you don't remember, please make your best guess.

	Month	Day	Year
Please Select:			

Has anyone in your household been told to quarantine, isolate, or limit time at their school or workplace since January 2020 because: they were sick or exposed to someone with COVID-19?

- Yes
- No
- I don't know
- Prefer not to answer

Did they follow the advice and isolate, quarantine, or stay away from their workplace or educational facility?

- ○ Yes
- ○ No
- ○ I don't know
- ○ Prefer not to answer

Participant Activities/Movement

How many times did you leave your home (or property, apartment) yesterday?

- ○ Did not leave
- ○ Once
- ○ 2 times
- ○ 3 times
- ○ 4 times
- ○ 5 times
- ○ 6 or more times
- ○ Prefer not to answer

Where did you go when you left your home? (Check all that apply)

- ☐ Another person's home
- ☐ A workplace
- ☐ A hospital, doctor's office, or other healthcare center
- ☐ Retail including grocery store, pharmacy, liquor store
- ☐ Church, Temple, or other place of worship
- ☐ A shared space in my building or residential compound
- ☐ A restaurant, bar, or cafe
- ☐ Small event < 10 people
- ☐ Medium size event 10-50 people
- ☐ Large event (music concert, sports game, movie, etc.)

- [ ] Park or other public space (including walking along a sidewalk)
- [ ] Other
- [ ] Prefer not to answer

What is the farthest distance that you went from your home yesterday?

- ○ Less than 1 kilometre
- ○ 1 to 10 kilometres
- ○ 10 to 100 kilometres
- ○ More than 100 kilometres
- ○ Prefer not to answer

How did you travel when you left your home? (Check all that apply)

- [ ] I only walked (I did not use other transportation)
- [ ] Bicycle, moped, or motorcycle
- [ ] Airplane
- [ ] Public transportation (e.g. bus, train, subway, tram, airplane etc)
- [ ] Alone in a car
- [ ] In a car with someone else (not a taxi)
- [ ] ____________ Other (please specify)
- [ ] Prefer not to answer

Did you use a face mask yesterday?

- ○ Yes
- ○ No
- ○ Prefer not to answer

Where did you use your face mask yesterday? (Check all that apply)

- ☐ Everywhere outside my house
- ☐ When walking on the street
- ☐ When cycling
- ☐ On public transport
- ☐ In supermarkets/shops
- ☐ In cinema/bar/restaurant
- ☐ At home
- ☐ At work/school/college/university
- ☐ ____________ Other (please specify)
- ☐ Prefer not to answer

Take your best guess for the total amount of time you wore a mask yesterday (hours and minutes)?

- ○ Less than 30mins
- ○ 30mins-59mins
- ○ 1hr-1h59mins
- ○ 2hrs-2hr59mins
- ○ 3hrs-3h59mins
- ○ More than 4hrs

In the last 3 hours, have you been in your home?

- ○ Yes
- ○ No
- ○ Prefer not to answer

In the last 3 hours, have many times did you wash your hands with soap?

- 0
- 1-3 times
- 3-6 times
- More than 6 times
- Prefer not to answer

In the last 3 hours, how many times did you use hand sanitizer?

- 0
- 1-3 times
- 3-6 times
- More than 6 times
- Prefer not to answer

Yesterday, which type of public transportation did you use? (please check all that apply)

- ☐ Airplane
- ☐ Bus
- ☐ Taxi, Uber, or similar ride-hailing app
- ☐ Train including skytrain
- ☐ ________ Other (please specify)
- ☐ Prefer not to answer

Yesterday, for about how long were you on public transportation?

- Less than 30 minutes
- 31 minutes to 1 hour
- 1 to 2 hours

- ○ 3 to 4 hours
- ○ 5 hours or more
- ○ Prefer not to answer

Yesterday, did you wear any of the following while on public transportation? (Please check all that apply)

- ☐ A face mask or other covering over your nose and mouth (e.g., face shield, bandana)
- ☐ Gloves
- ☐ Other protective equipment
- ☐ None of the above
- ☐ ________ Other (please describe)
- ☐ Prefer not to answer

Have you travelled outside Canada at all since Jan 2020? And if so, to where?

- ○ Yes, I travelled outside Canada (please write country/countries in the space below) ________
- ○ No, I have not traveled outside Canada

In-person Contact

*Now we would like to ask you some questions about people you had **in-person, face-to-face** contact with yesterday.*
*By **in-person, face-to-face contact**, we mean EITHER:*

A. An in-person two-way conversation with three or more words
OR
B. Physical skin-to-skin contact (for example, a handshake, hug, kiss, or contact sports).

This includes family members, friends, co-workers, people you spoke to in shops, bus drivers, strangers, etc... and people of ALL ages.
*Please **do not** count people you contacted only with things like telephone, text, or online.*

How many people did you have in-person contact with between 5 am yesterday and 5 am today?

0
○
1
○
2
○
3
○
4
○
5
○
6
○
7
○
8
○
9
○
10
○

11-15

○

16-20

○

21-25

○

26-30

○

31-35

○

36-40

○

41-45

○

46-50

○

51 or more

○

Prefer not to answer

○

Please add a non-identifying "nickname" for each of the people you had face-to-face or physical contact with (e.g., DG, checkout person, bus driver, child #2). This "nickname" will help you to answer questions about this contact.

1st person label ______________________

2nd person label	
3rd person label	
4th person label	
5th person label	
6th person label	
7th person label	
8th person label	
9th person label	
10th person label	

For the people you "nicknamed" and had **in-person** contact with between **5am yesterday and 5am today,** please use the drop-down arrow to answer the questions for each person (if no nickname is listed, leave row blank).

	I believe this person identifies as?	What is the age of this person (best guess)?
${q://QID265/ChoiceTextEntryValue/1}		
${q://QID265/ChoiceTextEntryValue/2}		
${q://QID265/ChoiceTextEntryValue/3}		
${q://QID265/ChoiceTextEntryValue/4}		
${q://QID265/ChoiceTextEntryValue/5}		
${q://QID265/ChoiceTextEntryValue/6}		
${q://QID265/ChoiceTextEntryValue/7}		
${q://QID265/ChoiceTextEntryValue/8}		
${q://QID265/ChoiceTextEntryValue/9}		
${q://QID265/ChoiceTextEntryValue/10}		

For the people you "nicknamed" and had **in-person** contact with between **5am yesterday and 5am today,** please use the drop-down arrow to answer the questions for each person (if no nickname is listed, leave row blank).

	What is the age of this person (best guess)?
${q://QID265/ChoiceTextEntryValue/1}	
${q://QID265/ChoiceTextEntryValue/2}	
${q://QID265/ChoiceTextEntryValue/3}	
${q://QID265/ChoiceTextEntryValue/4}	
${q://QID265/ChoiceTextEntryValue/5}	
${q://QID265/ChoiceTextEntryValue/6}	
${q://QID265/ChoiceTextEntryValue/7}	
${q://QID265/ChoiceTextEntryValue/8}	
${q://QID265/ChoiceTextEntryValue/9}	
${q://QID265/ChoiceTextEntryValue/10}	

For the people you "nicknamed" and had **in-person** contact with between **5am yesterday and 5am today,** please use the drop-down arrow to answer the questions for each person (if no nickname is listed, leave row blank).

	What was the smallest distance between you and the contact when talking?	About how lon[illegible] contact last?
${q://QID265/ChoiceTextEntryValue/1}		
${q://QID265/ChoiceTextEntryValue/2}		
${q://QID265/ChoiceTextEntryValue/3}		

${q://QID265/ChoiceTextEntryValue/4}		
${q://QID265/ChoiceTextEntryValue/5}		
${q://QID265/ChoiceTextEntryValue/6}		
${q://QID265/ChoiceTextEntryValue/7}		
${q://QID265/ChoiceTextEntryValue/8}		
${q://QID265/ChoiceTextEntryValue/9}		
${q://QID265/ChoiceTextEntryValue/10}		

For the people you "nicknamed" and had **in-person** contact with between **5am yesterday and 5am today,** please answer the following question (if no nickname is listed, leave row blank).

During the contact did you wear any of the following? Please check all that apply

	A face mask or any other covering (e.g., bandana)	Gloves	Other protective equipment	I did not wear protective equipment
${q://QID265/ChoiceTextEntryValue/1}	☐	☐	☐	☐
${q://QID265/ChoiceTextEntryValue/2}	☐	☐	☐	☐
${q://QID265/ChoiceTextEntryValue/3}	☐	☐	☐	☐
${q://QID265/ChoiceTextEntryValue/4}	☐	☐	☐	☐
${q://QID265/ChoiceTextEntryValue/5}	☐	☐	☐	☐
${q://QID265/ChoiceTextEntryValue/6}	☐	☐	☐	☐
${q://QID265/ChoiceTextEntryValue/7}	☐	☐	☐	☐

${q://QID265/ChoiceTextEntryValue/8}	☐	☐	☐	☐
${q://QID265/ChoiceTextEntryValue/9}	☐	☐	☐	☐
${q://QID265/ChoiceTextEntryValue/10}	☐	☐	☐	☐

When you had **in-person** contact **yesterday** (5am yesterday to 5am today) with "**${q://QID265/ChoiceTextEntryValue/1}**" , where did the contact take place?

If you had more than one in-person contact event with this person yesterday, please check all that apply.

- ☐ In my home
- ☐ In somebody else's home or home of person I had contact with
- ☐ In a store or business (grocery store, bookstore, etc.)
- ☐ In a restaurant or bar
- ☐ At school
- ☐ At work
- ☐ On the street
- ☐ In a place of worship (church, temple, synagogue, mosque, gudwara etc.)
- ☐ At a place of entertainment (e.g movie theatre, concert)
- ☐ At a place for sports such as a gym or sports club/match
- ☐ In transit/public transport (car, bus, subway, etc.)
- ☐ Outside, for example in a park, on the street or in the countryside
- ☐ ________ Other (please specify)
- ☐ Prefer not to answer
- ☐ Not Applicable

With "**${q://QID265/ChoiceTextEntryValue/2}**", when you had **in-person** contact **yesterday** (5am yesterday to 5am today), where did the contact take place?

If you had more than one in-person contact event with this person yesterday,

please check all that apply.

- [ ] In my home
- [ ] In somebody else's home
- [ ] In a store or business (grocery store, bookstore, etc.)
- [ ] In a restaurant or bar
- [ ] At school
- [ ] At work
- [ ] On the street
- [ ] In a place of worship (church, temple, synagogue, mosque, gudwara etc.)
- [ ] At a place of entertainment (e.g movie theatre, concert)
- [ ] At a place for sports such as a gym or sports club/match
- [ ] In transit/public transport (car, bus, subway, etc.)
- [ ] Outside, for example in a park, on the street or in the countryside
- [ ] ________ Other (please specify)
- [ ] Prefer not to answer
- [ ] Not Applicable

With "**${q://QID265/ChoiceTextEntryValue/3}**", when you had **in-person** contact **yesterday** (5am yesterday to 5am today), where did the contact take place?
If you had more than one in-person contact event with this person yesterday, please check all that apply.

- [ ] In your home
- [ ] In somebody else's home
- [ ] In a store or business (grocery store, bookstore, etc.)
- [ ] In a restaurant or bar
- [ ] At school
- [ ] At work
- [ ] On the street
- [ ] In a place of worship (church, temple, synagogue, mosque, gudwara etc.)

- [ ] At a place of entertainment (e.g movie theatre, concert)
- [ ] At a place for sports such as a gym or sports club/match
- [ ] In transit/public transport (car, bus, subway, etc.)
- [ ] Outside, for example in a park, on the street or in the countryside
- [ ] ________ Other (please specify)
- [ ] Prefer not to answer
- [ ] Not Applicable

With "**${q://QID265/ChoiceTextEntryValue/4}**", when you had **in-person** contact **yesterday** (5am yesterday to 5am today), where did the contact take place?
If you had more than one in-person contact event with this person yesterday, please check all that apply.

- [ ] In your home
- [ ] In somebody else's home
- [ ] In a store or business (grocery store, bookstore, etc.)
- [ ] In a restaurant or bar
- [ ] At school
- [ ] At work
- [ ] On the street
- [ ] In a place of worship (church, temple, synagogue, mosque, gudwara etc.)
- [ ] At a place of entertainment (e.g movie theatre, concert)
- [ ] At a place for sports such as a gym or sports club/match
- [ ] In transit/public transport (car, bus, subway, etc.)
- [ ] Outside, for example in a park, on the street or in the countryside
- [ ] ________ Other (please specify)
- [ ] Prefer not to answer
- [ ] Not Applicable

With "**${q://QID265/ChoiceTextEntryValue/5}**", when you had **in-person**

contact **yesterday** (5am yesterday to 5am today), where did the contact take place?
If you had more than one in-person contact event with this person yesterday, please check all that apply.

- ☐ In your home
- ☐ In somebody else's home
- ☐ In a store or business (grocery store, bookstore, etc.)
- ☐ In a restaurant or bar
- ☐ At school
- ☐ At work
- ☐ On the street
- ☐ In a place of worship (church, temple, synagogue, mosque, gudwara etc.)
- ☐ At a place of entertainment (e.g movie theatre, concert)
- ☐ At a place for sports such as a gym or sports club/match
- ☐ In transit/public transport (car, bus, subway, etc.)
- ☐ Outside, for example in a park, on the street or in the countryside
- ☐ ________ Other (please specify)
- ☐ Prefer not to answer
- ☐ Not Applicable

With **"${q://QID265/ChoiceTextEntryValue/6}"**, when you had **in-person** contact **yesterday** (5am yesterday to 5am today), where did the contact take place?
If you had more than one in-person contact event with this person yesterday, please check all that apply.

- ☐ In your home
- ☐ In somebody else's home
- ☐ In a store or business (grocery store, bookstore, etc.)
- ☐ In a restaurant or bar
- ☐ At school

- [ ] At work
- [ ] On the street
- [ ] In a place of worship (church, temple, synagogue, mosque, gudwara etc.)
- [ ] At a place of entertainment (e.g movie theatre, concert)
- [ ] At a place for sports such as a gym or sports club/match
- [ ] In transit/public transport (car, bus, subway, etc.)
- [ ] Outside, for example in a park, on the street or in the countryside
- [ ] ________ Other (please specify)
- [ ] Prefer not to answer
- [ ] Not Applicable

With "**${q://QID265/ChoiceTextEntryValue/7}**", when you had **in-person** contact **yesterday** (5am yesterday to 5am today), where did the contact take place?
If you had more than one in-person contact event with this person yesterday, please check all that apply.

- [ ] In your home
- [ ] In somebody else's home
- [ ] In a store or business (grocery store, bookstore, etc.)
- [ ] In a restaurant or bar
- [ ] At school
- [ ] At work
- [ ] On the street
- [ ] In a place of worship (church, temple, synagogue, mosque, gudwara etc.)
- [ ] At a place of entertainment (e.g movie theatre, concert)
- [ ] At a place for sports such as a gym or sports club/match
- [ ] In transit/public transport (car, bus, subway, etc.)
- [ ] Outside, for example in a park, on the street or in the countryside
- [ ] ________ Other (please specify)
- [ ] Prefer not to answer

- [ ] Not Applicable

With "**${q://QID265/ChoiceTextEntryValue/8}**", when you had **in-person** contact **yesterday** (5am yesterday to 5am today), where did the contact take place?
If you had more than one in-person contact event with this person yesterday, please check all that apply.

- [ ] In your home
- [ ] In somebody else's home
- [ ] In a store or business (grocery store, bookstore, etc.)
- [ ] In a restaurant or bar
- [ ] At school
- [ ] At work
- [ ] On the street
- [ ] In a place of worship (church, temple, synagogue, mosque, gudwara etc.)
- [ ] At a place of entertainment (e.g movie theatre, concert)
- [ ] At a place for sports such as a gym or sports club/match
- [ ] In transit/public transport (car, bus, subway, etc.)
- [ ] Outside, for example in a park, on the street or in the countryside
- [ ] ______ Other (please specify)
- [ ] Prefer not to answer
- [ ] Not Applicable

With "**${q://QID265/ChoiceTextEntryValue/9}**", when you had **in-person** contact **yesterday** (5am yesterday to 5am today), where did the contact take place?
If you had more than one in-person contact event with this person yesterday, please check all that apply.

- [ ] In your home

- [ ] In somebody else's home
- [ ] In a store or business (grocery store, bookstore, etc.)
- [ ] In a restaurant or bar
- [ ] At school
- [ ] At work
- [ ] On the street
- [ ] In a place of worship (church, temple, synagogue, mosque, gudwara etc.)
- [ ] At a place of entertainment (e.g movie theatre, concert)
- [ ] At a place for sports such as a gym or sports club/match
- [ ] In transit/public transport (car, bus, subway, etc.)
- [ ] Outside, for example in a park, on the street or in the countryside
- [ ] ________ Other (please specify)
- [ ] Prefer not to answer
- [ ] Not Applicable

With "**${q://QID265/ChoiceTextEntryValue/10}**", when you had **in-person** contact **yesterday** (5am yesterday to 5am today), where did the contact take place?
If you had more than one in-person contact event with this person yesterday, please check all that apply.

- [ ] In your home
- [ ] In somebody else's home
- [ ] In a store or business (grocery store, bookstore, etc.)
- [ ] In a restaurant or bar
- [ ] At school
- [ ] At work
- [ ] On the street
- [ ] In a place of worship (church, temple, synagogue, mosque, gudwara etc.)
- [ ] At a place of entertainment (e.g movie theatre, concert)
- [ ] At a place for sports such as a gym or sports club/match
- [ ] In transit/public transport (car, bus, subway, etc.)

- [ ] Outside, for example in a park, on the street or in the countryside
- [ ] ______ Other (please specify)
- [ ] Prefer not to answer
- [ ] Not Applicable

You said you had more than 10 in-person contacts. Where did **majority** of these contacts take place?

- ○ In my home
- ○ In other people's home
- ○ In a store (e.g grocery, bookstore, clothing, office supplies etc)
- ○ In a place that serves food (e.g. restaurant, bar, coffee shop
- ○ At a school (e.g., elementary, high school, university, college)
- ○ At my workplace or other person's workplace
- ○ In a place of worship (church, temple, synagogue, mosque, gudwara etc.)
- ○ At a place of entertainment (e.g., movie theatre, concert)
- ○ At a place for sports (e.g., gym, sports club)
- ○ In transit/public transport (e.g., car, bus, subway)
- ○ Outside (e.g., in a park, on the street.in the country)
- ○ ______ Other (please specify)
- ○ Prefer not to answer
- ○ Not Applicable

Which of these best describes your work/occupation or the other person's workplace where these contacts took place?

- ○ Food and beverage servers
- ○ Registered nurses and registered psychiatric nurses
- ○ Cashiers at food and beverage, retail stores etc
- ○ Nurse aides, orderlies and patient service associates
- ○ Hairstylists and barbers

- Firefighters
- Pharmacists
- Home support workers, housekeepers and related occupations
- Paramedical occupations & Allied primary health practitioners
- physicians
- Pharmacists
- Dentists, Dental hygienists and dental therapists
- Pursers and flight attendants
- Physiotherapists
- Medical administrative assistants
- Social workers
- Medical laboratory technicians and pathologists' assistants
- ______ Other

You said you had more than 10 in-person contacts. What was the age-group for most of these contacts you interacted with?

- Less than 2 years
- 2-5 years
- 6-17 years
- 18-24 years
- 25-34 years
- 35-44 years
- 45-54 years
- 55-64 years
- 65-74 years
- 75 or greater
- All ages
- Prefer not to answer
- I don't know

You said you had more than 10 in-person contacts. For most of these contacts, about how long did each contact last?

- Less than a minute
- 1 to 15minutes
- 15 minutes to an hour
- 1-2 hours
- 3-4 hours
- 5-6hours
- 7 or more hours
- Prefer not to answer

Attitude towards COVID-19

How much do you know about COVID-19?

- Have never heard of it
- Have heard of it, but that's about it
- Know a little about it
- Know a fair amount about it
- Know a lot about it
- Prefer not to answer

To what extent do you agree or disagree with the following statements?

	Strongly agree	Agree	Neither agree nor disagree	Disagree	Strongly disagree	Prefer not to answer

COVID-19 would be a serious illness for me if I caught it.	○	○	○	○	○	○
I think I am likely to catch COVID-19	○	○	○	○	○	○
If I don’t follow government advice, I might spread COVID-19 to someone who is vulnerable.	○	○	○	○	○	○

How effective do you think the following are at slowing the spread of COVID-19?

	Very effective	Fairly effective	Not very effective	Not at all effective	I don't know/Prefer not to answer
Meeting up with fewer people than normal	○	○	○	○	○
Avoiding crowded spaces	○	○	○	○	○

How effective do you think the following is at slowing the spread of COVID-19?

Staying at home for 14 days if <u>you</u> have ...

	Very effective	Fairly effective	Not very effective	Not at all effective	I don't know/Prefer not to answer
**Mild** symptoms (e.g., mild cough)	○	○	○	○	○

**Severe** symptoms (e.g., severe cough or high temperature)	○	○	○	○	○

How effective do you think the following is at slowing the spread of COVID-19?

Staying at home for 14 days if anyone other than yourself in your household has ..

	Very effective	Fairly effective	Not very effective	Not at all effective	I don't know/Prefer not to answer
**Mild** symptoms (e.g., mild cough)	○	○	○	○	○
**Severe** symptoms (e.g., severe cough or high temperature)	○	○	○	○	○

If you wanted to, how confident are you that you could...

	Not very confident	Only a little confident	Fairly confident	Very confident	I don't know/Prefer not to answer
Avoid crowded places	○	○	○	○	○
Not use public transportation	○	○	○	○	○
Meet up with fewer people than normal?	○	○	○	○	○

If you wanted to, how confident are you that you could stay at home for 14 days if you have...

	Not very confident	Only a little confident	Fairly confident	Very confident	I don't know/Prefer not to answer
**Mild** symptoms (e.g., mild cough)?	○	○	○	○	○
**Severe** cough or a high temperature	○	○	○	○	○

If you wanted to, how confident are you that you could stay at home for 14 days if someone else in your household has...

	Not very confident	Only a little confident	Fairly confident	Very confident	I don't know/Prefer not to answer
**Mild** symptoms such as a **mild** cough	○	○	○	○	○
**Severe** symptoms such as a severe cough or a high temperature	○	○	○	○	○

To what extent do you agree or disagree with the statements below?

	Strongly agree	Tend to agree	Neither agree nor disagree	Tend to disagree	Strongly disagree	Prefer not to answer	Not applicable/don' know

My boss expects me to work when I am feeling unwell or sick	○	○	○	○	○	○	○
If I could not work because of COVID-19, I would still get paid	○	○	○	○	○	○	○

To what extent do you agree or disagree with the following?

If I had to isolate myself for 14 days because of COVID-19...

	Strongly agree	Tend to agree	Neither agree nor disagree	Tend to disagree	Strongly disagree	Prefer not to answer	Not applicable
I would have enough food and supplies for 14 days	○	○	○	○	○	○	○

Someone else would be able to look after my children	○	○	○	○	○	○	○

How much, if at all, have you changed the number of face-to-face interaction with other people as a result of the COVID19 pandemic?

- ○ I have greatly reduced face-to-face interaction with others
- ○ I have somewhat reduced face-to-face interaction with others
- ○ I have not changed my interactions with others
- ○ I have increased face-to-face interaction with others
- ○ Prefer not to answer

How well do you think you are doing at keeping physically distanced from people outside your home?

- ○ Very well
- ○ Somewhat well
- ○ Neutral
- ○ Somewhat poorly
- ○ Very poorly
- ○ Prefer not to answer

How concerned are you personally about the spread of COVID19?

- ○ Very concerned
- ○ Somewhat concerned
- ○ Not very concerned
- ○ Not at all concerned

- Prefer not to answer

Internal and Social Media Use

About how often do you use the internet?

- Almost all day
- Several times a day
- About once a day
- Several times a week
- Less often
- Never
- Prefer not to answer

Do you ever use social media sites like Facebook, Twitter, or Instagram?

- Yes
- No
- Prefer not to answer

Thinking about the social media sites that you use; about how often do you visit or use each of the following?

	Several times a day	A few times a week	Every few weeks	Less often	Never	Prefer not to answer
Instagram	○	○	○	○	○	○
Facebook	○	○	○	○	○	○
Twitter	○	○	○	○	○	○

Snapchat	○	○	○	○	○	○
Youtube	○	○	○	○	○	○

Demographics #2

What do you identify as?

- ○ Man
- ○ Woman
- ○ Transgender
- ○ Gender-neutral
- ○ Agender
- ○ Pangender
- ○ Genderqueer
- ○ Two-spirit
- ○ __________ Other
- ○ Prefer not to answer

What is the highest level of school you have completed or the highest degree you have received?

- ○ Less than high school degree
- ○ High school graduate (high school diploma or equivalent including GED)
- ○ Some college/university but no degree
- ○ Associate degree or diploma in college/university (2-year)
- ○ Bachelor's degree in college (4-year)
- ○ Master's degree
- ○ Doctoral degree
- ○ Professional degree (e.g., JD, MD)
- ○ Prefer not to answer

What is your current employment status?

- ○ Employed full-time (30 hours or more/week)
- ○ Employed part-time (less than 30 hours/week)
- ○ Self-employed
- ○ Unemployed but looking for a job
- ○ Unemployed and not looking for a job
- ○ Full-time parent, homemaker
- ○ Retired
- ○ Student/Pupil
- ○ Long-term sick or disabled
- ○ Prefer not to answer

As of March 2020, what occupation or industry have you most often worked in?

- ○ I do not work
- ○ Business, finance and administration occupations
- ○ Health occupations (e.g., medical, social work, psychology)
- ○ Management occupations
- ○ Natural and applied sciences and related occupations
- ○ Natural resources, agriculture and related production occupations
- ○ Occupations in art, culture, recreation and sport
- ○ Occupations in education, law and social, community and government services
- ○ Occupations in manufacturing and utilities
- ○ Sales and service occupations
- ○ Trades, transport and equipment operators and related occupations
- ○ ________ Other (prefer to self describe)
- ○ Prefer not to answer

Where do you most often work?

- ◯ Home - Indoors
- ◯ Home - Outdoors (e.g., farm)
- ◯ On work site (e.g., business, school, retail, local construction)
- ◯ On work site, but away from home (e.g., construction site away from hometown)
- ◯ Prefer not to answer

What is your postal code? (e.g V6T 1Z4)

[ ]

Future Contact for Survey

To help improve public health, we study how interaction patterns change over time. We will be asking some people to answer a similar but shorter survey in a few weeks. These surveys are very important for planning British Columbia's response to COVID-19! Participation is optional, and you can request the deletion of your email address at any time. This follow up survey will take less than 5 minutes to complete. May we contact you again for a similar survey? If you agree to be contacted again, the next question will ask for your contact information. If you choose not to be contacted again, your responses on this survey will be completely anonymous and we won't contact you again.

- ◯ Yes, I agree to be contacted again
- ◯ No, do not contact me about this again

Please provide the following information

First name	
Last name	
E-mail	

Telephone [ ]

I consent to combining of my survey responses with health care utilization data, if yes please provide your...

- ○ [ ] Personal health number
- ○ I do not consent to combining of my survey responses with health care utilization data

Would you like to be entered in a draw to win a $25 gift card?

- ○ Yes
- ○ No, I do not want to enter the draw

Dr Naveed Janjua, Principal Investigator
COVID-19- BC Population Mixing Patterns Survey Team
BC Centre for Disease Control
Email: data_analytics@bccdc.ca

Powered by Qualtrics
