# Supplementary material for: Ethnic Disparities in COVID-19 Vaccine Mistrust and Receipt in British Columbia, Canada: Population Survey
Source: JMIR Public Health Surveill. 2024 Feb 16;10:e48466. doi: 10.2196/48466 (PMC10896316; doi:10.2196/48466)
Supplement: Multimedia Appendix 2 [file publichealth_v10i1e48466_app2.pdf]

BCMIX- COVID-19 FOLLOW UP SURVEY

Survey linkage Info

Welcome to the BCMix Follow-up Survey

Please provide the following information

First name	
Last name	
E-mail	

COVID-19 Diagnosis

Since the last time you completed the survey, have you had any of the following symptoms? Check all that apply

- ☐ I have not had any symptoms
- ☐ Headache
- ☐ Fever
- ☐ Stuffy nose/ congestion
- ☐ Loss of smell or taste
- ☐ New or worsening cough
- ☐ Difficulty breathing/shortness of breath
- ☐ Confusion
- ☐ Vomiting
- ☐ Chills
- ☐ Weakness
- ☐ Muscle pain
- ☐ Fatigue
- ☐ Nausea
- ☐ Diarrhea
- ☐ ________ Other; please specify

- [ ] I don't know
- [ ] Prefer not to answer

When did your first symptom start? If you do not remember, enter your best guess.

	Month	Day	Year
Please select:			

Have you done any of the following for these symptoms? (please check all that apply)

- [ ] Called 811
- [ ] Called Family doctor/ GP
- [ ] Consulted family doc through telehealth
- [ ] Visited, Family doctor's /GP office
- [ ] Visited, Community/public health clinic
- [ ] Visited, hospital emergency department
- [ ] Visited, urgent care clinic
- [ ] Visited, COVID testing centre
- [ ] Been admitted to hospital
- [ ] Used home remedies
- [ ] Treated symptoms with over the counter medications (Tylenol, etc.)
- [ ] ______ Others; please specify
- [ ] None of the above
- [ ] Prefer not to answer

Before these symptoms, had you been in close contact with anyone who **either**: **(A)** had any of those symptoms [fever, new or worsening cough, headache, chills, weakness, muscle pain, stuffy nose/congestion, sore throat, difficulty breathing/shortness of breath, nausea, diarrhea, fatigue, loss of smell or taste, confusion, vomiting]; **OR (B)** was diagnosed positive for COVID-19 within 14 days before you felt sick?

- ○ Yes
- ○ No
- ○ I don't know
- ○ Prefer not to answer

Did you isolate, or stay away from your workplace or educational facility?

- Yes
- No
- I don't know
- Prefer not to answer

Have you been tested for COVID-19?

- Yes
- No
- Prefer not to answer

Did you test positive for COVID-19?

- Yes
- No
- I don't know/Prefer not to answer

Has anyone in your household **either**: **(A)** had any of the following symptoms: fever, new or worsening cough, headache, chills, weakness, muscle pain, stuffy nose/congestion, sore throat, difficulty breathing/shortness of breath, nausea, diarrhea, fatigue, loss of smell or taste, confusion, vomiting; **OR (B)** tested positive for COVID-19 since January 2020?

- Yes
- No
- I don't know
- Prefer not to answer

When did **their** first symptom start? If you don’t remember, please make your best guess.

	Month	Day	Year
Please Select:			

Has anyone in your household been told to quarantine, isolate, or limit time at their school or workplace since January 2020 because: they were sick or exposed to someone with COVID-19?

- Yes
- No
- I don't know
- Prefer not to answer

Did they follow the advice and isolate, quarantine, or stay away from their workplace or educational facility?

- Yes
- No
- I don't know
- Prefer not to answer

Vaccination

Have you received the COVID-19 vaccine (either 1st or 2nd shot)?

- Yes
- No

Indicate your level of agreement with the following statements.

I believe I am at risk of becoming infected with COVID-19.

- 1-Strongly Disagree
- 2-Disagree
- 3-Neutral
- 4-Agree
- 5-Strongly Agree

With the way my life is, I believe I am at a high risk of getting COVID-19 (e.g. risks at my work, recreational activities, people I live with, etc.)

- 1-Strongly Disagree
- 2-Disagree
- 3-Neutral
- 4-Agree
- 5-Strongly Agree

I believe a COVID-19 Vaccine will protect me from getting the virus.

- 1-Strongly Disagree
- 2-Disagree
- 3-Neutral
- 4-Agree
- 5-Strongly Agree

I believe a COVID-19 vaccine will decrease my chance of getting seriously ill from COVID-19.

- 1-Strongly Disagree
- 2-Disagree
- 3-Neutral
- 4-Agree
- 5-Strongly Agree

I ***do not*** trust the COVID-19 vaccine.

- 1-Strongly Disagree
- 2-Disagree
- 3-Neutral
- 4-Agree
- 5-Strongly Agree

I am concerned about the effectiveness of the COVID-19 vaccination.

- 1-Strongly Disagree
- 2-Disagree
- 3-Neutral
- 4-Agree
- 5-Strongly Agree

I am concerned about the safety of the COVID-19 vaccination.

- 1-Strongly Disagree
- 2-Disagree
- 3-Neutral
- 4-Agree
- 5-Strongly Agree

Most of the people I know are getting or have received the COVID-19 vaccine.

- 1-Strongly Disagree
- 2-Disagree
- 3- Don't know
- 4-Agree
- 5-Strongly Agree

Most of the people who are important to me (my family, relatives and/or friends) think I should get the COVID-19 vaccine.

- 1-Strongly Disagree
- 2-Disagree
- 3-Don't know
- 4-Agree
- 5-Strongly Agree

If I choose to get the COVID-19 vaccine, I believe it will be easy to get it.

- 1-Strongly Disagree

- 2-Disagree
- 3-Don't know
- 4-Agree
- 5-Strongly Agree

I plan to get the COVID-19 vaccine.

- 1-Strongly Disagree
- 2-Disagree
- 3-Undecided
- 4-Agree
- 5-Strongly Agree

Participant Activities/Movement

How many times did you leave your home (or property, apartment) yesterday?

- Did not leave
- Once
- 2 times
- 3 times
- 4 times
- 5 times
- 6 or more times
- Prefer not to answer

Where did you go when you left your home? (Check all that apply)

- Another person's home
- A workplace
- A hospital, doctor's office, or other healthcare center
- Retail including grocery store, pharmacy, liquor store
- Church, Temple, or other place of worship
- A shared space in my building or residential compound
- A restaurant, bar, or cafe

- ☐ Small event < 10 people
- ☐ Medium size event 10-50 people
- ☐ Large event (music concert, sports game, movie, etc.)
- ☐ Park or other public space (including walking along a sidewalk)
- ☐ Other
- ☐ Prefer not to answer

What is the farthest distance that you went from your home yesterday?

- ○ Less than 1 kilometre
- ○ 1 to 10 kilometres
- ○ 10 to 100 kilometres
- ○ More than 100 kilometres
- ○ Prefer not to answer

How did you travel when you left your home? (Check all that apply)

- ☐ I only walked (I did not use other transportation)
- ☐ Bicycle, moped, or motorcycle
- ☐ Airplane
- ☐ Public transportation (e.g. bus, train, subway, tram, airplane etc)
- ☐ Alone in a car
- ☐ In a car with someone else (not a taxi)
- ☐ ______ Other (please specify)
- ☐ Prefer not to answer

Did you use a face mask yesterday?

- ○ Yes
- ○ No
- ○ Prefer not to answer

Where did you use your face mask yesterday? (Check all that apply)

- ☐ Everywhere outside my house

- [ ] When walking on the street
- [ ] When cycling
- [ ] On public transport
- [ ] In supermarkets/shops
- [ ] In cinema/bar/restaurant
- [ ] At home
- [ ] At work/school/college/university
- [ ] ______ Other (please specify)
- [ ] Prefer not to answer

Take your best guess for the total amount of time you wore a mask yesterday (hours and minutes)?

- Less than 30mins
- 30mins-59mins
- 1hr-1h59mins
- 2hrs-2hr59mins
- 3hrs-3h59mins
- More than 4hrs

In the last 3 hours, have you been in your home?

- Yes
- No
- Prefer not to answer

In the last 3 hours, how many times did you wash your hands with soap?

- 0
- 1-3 times
- 3-6 times
- More than 6 times
- Prefer not to answer

In the last 3 hours, how many times did you use hand sanitizer?

- 0
- 1-3 times
- 3-6 times
- More than 6 times
- Prefer not to answer

Yesterday, which type of public transportation did you use? (please check all that apply)

- Airplane
- Bus
- Taxi, Uber, or similar ride-hailing app
- Train including skytrain
- ________ Other (please specify)
- Prefer not to answer

Yesterday, for about how long were you on public transportation?

- Less than 30 minutes
- 31 minutes to 1 hour
- 1 to 2 hours
- 3 to 4 hours
- 5 hours or more
- Prefer not to answer

Yesterday, did you wear any of the following while on public transportation? (Please check all that apply)

- A face mask or other covering over your nose and mouth (e.g., face shield, bandana)
- Gloves
- Other protective equipment
- None of the above
- ________ Other (please describe)
- Prefer not to answer

Have you travelled outside Canada at all since since the last time you completed the survey? And if so, to where?

- Yes, I travelled outside Canada (please write country/countries in the space below) [ ]
- No, I have not traveled outside Canada

In-person Contact

Now we would like to ask you some questions about people you had ***in-person, face-to-face*** *contact with yesterday.*
By ***in-person, face-to-face contact****, we mean EITHER:*

A. An in-person two-way conversation with three or more words
OR
B. Physical skin-to-skin contact (for example, a handshake, hug, kiss, or contact sports).

This includes family members, friends, co-workers, people you spoke to in shops, bus drivers, strangers, etc... and people of ALL ages.
Please ***do not*** *count people you contacted only with things like telephone, text, or online.*

How many people did you have in-person contact with between 5 am yesterday and 5 am today?

0	1	2	3	4	5	6	7	8	9	10	11-15	16-20	21-25	26-30	31-35	36-40	41-45	46-50	51 or more	Prefer not to answer
○	○	○	○	○	○	○	○	○	○	○	○	○	○	○	○	○	○	○	○	○

Please add a non-identifying "nickname" for each of the people you had face-to-face or physical contact with (e.g., DG, checkout person, bus driver, child #2). This "nickname" will help you to answer questions about this contact.

1st person label [ ]
2nd person label [ ]
3rd person label [ ]
4th person label [ ]
5th person label [ ]
6th person label [ ]
7th person label

8th person label	
9th person label	
10th person label	

For the people you "nicknamed" and had in-person contact with between 5am yesterday and 5am today, please use the drop-down arrow to answer the questions for each person (if no nickname is listed, leave row blank).

	I believe this person identifies as?	What is the age of this person (best guess)?	What is you
${q://QID267/ChoiceTextEntryValue/1}			
${q://QID267/ChoiceTextEntryValue/2}			
${q://QID267/ChoiceTextEntryValue/3}			
${q://QID267/ChoiceTextEntryValue/4}			
${q://QID267/ChoiceTextEntryValue/5}			
${q://QID267/ChoiceTextEntryValue/6}			
${q://QID267/ChoiceTextEntryValue/7}			
${q://QID267/ChoiceTextEntryValue/8}			
${q://QID267/ChoiceTextEntryValue/9}			
${q://QID267/ChoiceTextEntryValue/10}			

For the people you "nicknamed" and had **in-person** contact with between **5am yesterday and 5am today,** please use the drop-down arrow to answer the questions for each person (if no nickname is listed, leave row blank).

	What was the smallest distance between you and the contact when talking?	About how long did the in-person contact last?	**B** yo w
${q://QID267/ChoiceTextEntryValue/1}			
${q://QID267/ChoiceTextEntryValue/2}			
${q://QID267/ChoiceTextEntryValue/3}			
${q://QID267/ChoiceTextEntryValue/4}			
${q://QID267/ChoiceTextEntryValue/5}			
${q://QID267/ChoiceTextEntryValue/6}			
${q://QID267/ChoiceTextEntryValue/7}			
${q://QID267/ChoiceTextEntryValue/8}			

	What was the smallest distance between you and the contact when talking?	About how long did the in-person contact last?	B yo w
${q://QID267/ChoiceTextEntryValue/9}			
${q://QID267/ChoiceTextEntryValue/10}			

For the people you "nicknamed" and had **in-person** contact with between **5am yesterday and 5am today,** please answer the following question (if no nickname is listed, leave row blank).

During the contact did you wear any of the following? Please check all that apply.

	A face mask or any other covering (e.g., bandana)	Gloves	Other protective equipment	I did not wear protective equipment	Prefer not to answer
${q://QID267/ChoiceTextEntryValue/1}	☐	☐	☐	☐	☐
${q://QID267/ChoiceTextEntryValue/2}	☐	☐	☐	☐	☐
${q://QID267/ChoiceTextEntryValue/3}	☐	☐	☐	☐	☐
${q://QID267/ChoiceTextEntryValue/4}	☐	☐	☐	☐	☐
${q://QID267/ChoiceTextEntryValue/5}	☐	☐	☐	☐	☐
${q://QID267/ChoiceTextEntryValue/6}	☐	☐	☐	☐	☐
${q://QID267/ChoiceTextEntryValue/7}	☐	☐	☐	☐	☐
${q://QID267/ChoiceTextEntryValue/8}	☐	☐	☐	☐	☐
${q://QID267/ChoiceTextEntryValue/9}	☐	☐	☐	☐	☐
${q://QID267/ChoiceTextEntryValue/10}	☐	☐	☐	☐	☐

With **"${q://QID267/ChoiceTextEntryValue/1}"**, when you had **in-person** contact **yesterday** (5am yesterday to 5am today), where did the contact take place?

- ☐ In my home
- ☐ In somebody else's home or home of person I had contact with
- ☐ In a store or business (grocery store, bookstore, etc.)
- ☐ In a restaurant or bar
- ☐ At school

- [ ] At work
- [ ] On the street
- [ ] In a place of worship (church, temple, synagogue, msque, gudwara etc.)
- [ ] At a place of entertainment (e.g movie theatre, concert)
- [ ] At a place for sports such as a gym or sports club/match
- [ ] In transit/public transport (car, bus, subway, etc.)
- [ ] Outside, for example in a park, on the street or in the countryside
- [ ] ________ Other (please specify)
- [ ] Prefer not to answer
- [ ] Not Applicable

With "**${q://QID267/ChoiceTextEntryValue/2}**", when you had **in-person** contact **yesterday** (5am yesterday to 5am today), where did the contact take place?

- [ ] In my home
- [ ] In somebody else's home
- [ ] In a store or business (grocery store, bookstore, etc.)
- [ ] In a restaurant or bar
- [ ] At school
- [ ] At work
- [ ] On the street
- [ ] In a place of worship (church, temple, synagogue, mosque, gudwara etc.)
- [ ] At a place of entertainment (e.g movie theatre, concert)
- [ ] At a place for sports such as a gym or sports club/match
- [ ] In transit/public transport (car, bus, subway, etc.)
- [ ] Outside, for example in a park, on the street or in the countryside
- [ ] ________ Other (please specify)
- [ ] Prefer not to answer
- [ ] Not Applicable

With "**${q://QID267/ChoiceTextEntryValue/3}**", when you had **in-person** contact **yesterday** (5am yesterday to 5am today), where did the contact take place?

- ☐ In your home
- ☐ In somebody else's home
- ☐ In a store or business (grocery store, bookstore, etc.)
- ☐ In a restaurant or bar
- ☐ At school
- ☐ At work
- ☐ On the street
- ☐ In a place of worship (church, temple, synagogue, mosque, gudwara etc.)
- ☐ At a place of entertainment (e.g movie theatre, concert)
- ☐ At a place for sports such as a gym or sports club/match
- ☐ In transit/public transport (car, bus, subway, etc.)
- ☐ Outside, for example in a park, on the street or in the countryside
- ☐ ________ Other (please specify)
- ☐ Prefer not to answer
- ☐ Not Applicable

With **"${q://QID267/ChoiceTextEntryValue/4}",** when you had **in-person** contact **yesterday** (5am yesterday to 5am today), where did the contact take place?

- ☐ In your home
- ☐ In somebody else's home
- ☐ In a store or business (grocery store, bookstore, etc.)
- ☐ In a restaurant or bar
- ☐ At school
- ☐ At work
- ☐ On the street
- ☐ In a place of worship (church, temple, synagogue, mosque, gudwara etc.)
- ☐ At a place of entertainment (e.g movie theatre, concert)
- ☐ At a place for sports such as a gym or sports club/match
- ☐ In transit/public transport (car, bus, subway, etc.)
- ☐ Outside, for example in a park, on the street or in the countryside
- ☐ ________ Other (please specify)
- ☐ Prefer not to answer

- [ ] Not Applicable

With "**${q://QID267/ChoiceTextEntryValue/5}**", when you had **in-person** contact **yesterday** (5am yesterday to 5am today), where did the contact take place?

- [ ] In your home
- [ ] In somebody else's home
- [ ] In a store or business (grocery store, bookstore, etc.)
- [ ] In a restaurant or bar
- [ ] At school
- [ ] At work
- [ ] On the street
- [ ] In a place of worship (church, temple, synagogue, mosque, gudwara etc.)
- [ ] At a place of entertainment (e.g movie theatre, concert)
- [ ] At a place for sports such as a gym or sports club/match
- [ ] In transit/public transport (car, bus, subway, etc.)
- [ ] Outside, for example in a park, on the street or in the countryside
- [ ] ________ Other (please specify)
- [ ] Prefer not to answer
- [ ] Not Applicable

With "**${q://QID267/ChoiceTextEntryValue/6}**", when you had **in-person** contact **yesterday** (5am yesterday to 5am today), where did the contact take place?

- [ ] In your home
- [ ] In somebody else's home
- [ ] In a store or business (grocery store, bookstore, etc.)
- [ ] In a restaurant or bar
- [ ] At school
- [ ] At work
- [ ] On the street
- [ ] In a place of worship (church, temple, synagogue, mosque, gudwara etc.)
- [ ] At a place of entertainment (e.g movie theatre, concert)

- [ ] At a place for sports such as a gym or sports club/match
- [ ] In transit/public transport (car, bus, subway, etc.)
- [ ] Outside, for example in a park, on the street or in the countryside
- [ ] ______ Other (please specify)
- [ ] Prefer not to answer
- [ ] Not Applicable

With "**${q://QID267/ChoiceTextEntryValue/7}**", when you had **in-person** contact **yesterday** (5am yesterday to 5am today), where did the contact take place?

- [ ] In your home
- [ ] In somebody else's home
- [ ] In a store or business (grocery store, bookstore, etc.)
- [ ] In a restaurant or bar
- [ ] At school
- [ ] At work
- [ ] On the street
- [ ] In a place of worship (church, temple, synagogue, mosque, gudwara etc.)
- [ ] At a place of entertainment (e.g movie theatre, concert)
- [ ] At a place for sports such as a gym or sports club/match
- [ ] In transit/public transport (car, bus, subway, etc.)
- [ ] Outside, for example in a park, on the street or in the countryside
- [ ] ______ Other (please specify)
- [ ] Prefer not to answer
- [ ] Not Applicable

With "**${q://QID267/ChoiceTextEntryValue/8}**", when you had **in-person** contact **yesterday** (5am yesterday to 5am today), where did the contact take place?

- [ ] In your home
- [ ] In somebody else's home
- [ ] In a store or business (grocery store, bookstore, etc.)
- [ ] In a restaurant or bar

- ☐ At school
- ☐ At work
- ☐ On the street
- ☐ In a place of worship (church, temple, synagogue, mosque, gudwara etc.)
- ☐ At a place of entertainment (e.g movie theatre, concert)
- ☐ At a place for sports such as a gym or sports club/match
- ☐ In transit/public transport (car, bus, subway, etc.)
- ☐ Outside, for example in a park, on the street or in the countryside
- ☐ ________ Other (please specify)
- ☐ Prefer not to answer
- ☐ Not Applicable

With "**${q://QID267/ChoiceTextEntryValue/9}**", when you had **in-person** contact **yesterday** (5am yesterday to 5am today), where did the contact take place?

- ☐ In your home
- ☐ In somebody else's home
- ☐ In a store or business (grocery store, bookstore, etc.)
- ☐ In a restaurant or bar
- ☐ At school
- ☐ At work
- ☐ On the street
- ☐ In a place of worship (church, temple, synagogue, mosque, gudwara etc.)
- ☐ At a place of entertainment (e.g movie theatre, concert)
- ☐ At a place for sports such as a gym or sports club/match
- ☐ In transit/public transport (car, bus, subway, etc.)
- ☐ Outside, for example in a park, on the street or in the countryside
- ☐ ________ Other (please specify)
- ☐ Prefer not to answer
- ☐ Not Applicable

With "**${q://QID267/ChoiceTextEntryValue/10}**", when you had **in-person** contact **yesterday** (5am yesterday to 5am today), where did the contact take

place?

- ☐ In your home
- ☐ In somebody else's home
- ☐ In a store or business (grocery store, bookstore, etc.)
- ☐ In a restaurant or bar
- ☐ At school
- ☐ At work
- ☐ On the street
- ☐ In a place of worship (church, temple, synagogue, mosque, gudwara etc.)
- ☐ At a place of entertainment (e.g movie theatre, concert)
- ☐ At a place for sports such as a gym or sports club/match
- ☐ In transit/public transport (car, bus, subway, etc.)
- ☐ Outside, for example in a park, on the street or in the countryside
- ☐ ________ Other (please specify)
- ☐ Prefer not to answer
- ☐ Not Applicable

You said you had more than 10 in-person contacts. Where did **majority** of these contacts take place?

- ○ In my home
- ○ In other people's home
- ○ In a store (e.g grocery, bookstore, clothing, office supplies etc)
- ○ In a place that serves food (e.g. restaurant, bar, coffee shop
- ○ At a school (e.g., elementary, high school, university, college)
- ○ At my workplace or other person’s workplace
- ○ In a place of worship (church, temple, synagogue, mosque, gudwara etc.)
- ○ At a place of entertainment (e.g., movie theatre, concert)
- ○ At a place for sports (e.g., gym, sports club)
- ○ In transit/public transport (e.g., car, bus, subway)
- ○ Outside (e.g., in a park, on the street.in the country)
- ○ ________ Other (please specify)
- ○ Prefer not to answer
- ○ Not Applicable

Which of these best describes your work/occupation or the other person's workplace where these contacts took place?

- Food and beverage servers
- Registered nurses and registered psychiatric nurses
- Cashiers at food and beverage, retail stores etc
- Nurse aides, orderlies and patient service associates
- Hairstylists and barbers
- Firefighters
- Pharmacists
- Home support workers, housekeepers and related occupations
- Paramedical occupations & Allied primary health practitioners
- Physicians
- Pharmacists
- Dentists, Dental hygienists and dental therapists
- Pursers and flight attendants
- Physiotherapists
- Medical administrative assistants
- Social workers
- Medical laboratory technicians and pathologists' assistants
- ______ Other

You said you had more than 10 in-person contacts. What was the age-group for most of these contacts you interacted with?

- Less than 2 years
- 2-5 years
- 6-17 years
- 18-24 years
- 25-34 years
- 35-44 years
- 45-54 years
- 55-64 years
- 65-74 years

- 75 or greater
- All ages
- Prefer not to answer
- I don't know

You said you had more than 10 in-person contacts. For most of these contacts, about how long did each contact last?

- Less than a minute
- 1 to 15minutes
- 15 minutes to an hour
- 1-2 hours
- 3-4 hours
- 5-6hours
- 7 or more hours
- Prefer not to answer

Future Contact for Survey

To help improve public health, we study how interaction patterns change over time. We may be contacting you again to complete this short survey in a few weeks. These surveys are very important for planning British Columbia's response to COVID-19! Participation is optional, and you can request the deletion of your email address at any time. May we contact you again?

- Yes, I agree to be contacted again
- No, do not contact me about this again

Dr Naveed Janjua, Principal Investigator
COVID-19- BC Population Mixing Patterns Survey Team
BC Centre for Disease Control
Email: data_analytics@bccdc.ca

Powered by Qualtrics
