# Supplementary material for: Ethnic Disparities in COVID-19 Vaccine Mistrust and Receipt in British Columbia, Canada: Population Survey
Source: JMIR Public Health Surveill. 2024 Feb 16;10:e48466. doi: 10.2196/48466 (PMC10896316; doi:10.2196/48466)
Supplement: Multimedia Appendix 3 [file publichealth_v10i1e48466_app3.docx]

**Multimedia Appendix 3:** Mistrust in COVID-19 vaccine by ethnicity in British Columbia.

|  | South Asian | | | Chinese | | | White | | | Other Ethnicity | | | All | | |
| --- | --- | --- | --- | --- | --- | --- | --- | --- | --- | --- | --- | --- | --- | --- | --- |
|  | N | % | 95% CI | N | % | 95% CI | N | % | 95% CI | N | % | 95% CI | N | % | 95% CI |
| COVID-19 Vaccine mistrust |  |  |  |  |  |  |  |  |  |  |  |  |  |  |  |
| Yes | 239 | 8.2 | (5.4, 11.1) | 223 | 7.1 | (4.6, 9.6) | 2,769 | 15.4 | (14.5, 16.2) | 707 | 15.2 | (13.1, 17.4) | 3,938 | 13.7 | (13.0, 14.5) |
| No | 2,276 | 78.3 | (73.8, 82.8) | 2,678 | 84.9 | (81.7, 88.0) | 13,499 | 75.0 | (74.1, 76.0) | 3,107 | 67.0 | (64.4, 69.5) | 21,560 | 75.1 | (74.2, 76.1) |
| Undecided | 391 | 13.5 | (9.6, 17.3) | 255 | 8.1 | (5.9, 10.2) | 1,724 | 9.6 | (8.9, 10.2) | 826 | 17.8 | (15.8, 19.8) | 3,197 | 11.1 | (10.4, 11.8) |
